# Rgs4 is a regulator of mTOR activity required for motoneuron axon outgrowth and neuronal development in zebrafish

**DOI:** 10.1038/s41598-021-92758-z

**Published:** 2021-06-25

**Authors:** Aya Mikdache, Marie-José Boueid, Lorijn van der Spek, Emilie Lesport, Brigitte Delespierre, Julien Loisel-Duwattez, Cindy Degerny, Marcel Tawk

**Affiliations:** 1 U1195, Inserm, University Paris-Saclay, 94276 Le Kremlin Bicêtre, France; 2 Ecole Polytechnique, University Paris-Saclay, 91120 Palaiseau, France; 3 Present Address: UMR 3215 – U934, Institut Curie, 75005 Paris, France

**Keywords:** Developmental biology, Neuroscience

## Abstract

The Regulator of G protein signaling 4 (Rgs4) is a member of the RGS proteins superfamily that modulates the activity of G-protein coupled receptors. It is mainly expressed in the nervous system and is linked to several neuronal signaling pathways; however, its role in neural development in vivo remains inconclusive. Here, we generated and characterized a *rgs4* loss of function model (*MZrgs4)* in zebrafish. *MZrgs4* embryos showed motility defects and presented reduced head and eye sizes, reflecting defective motoneurons axon outgrowth and a significant decrease in the number of neurons in the central and peripheral nervous system. Forcing the expression of Rgs4 specifically within motoneurons rescued their early defective outgrowth in *MZrgs4* embryos, indicating an autonomous role for Rgs4 in motoneurons. We also analyzed the role of Akt, Erk and mechanistic target of rapamycin (mTOR) signaling cascades and showed a requirement for these pathways in motoneurons axon outgrowth and neuronal development. Drawing on pharmacological and rescue experiments in *MZrgs4*, we provide evidence that Rgs4 facilitates signaling mediated by Akt, Erk and mTOR in order to drive axon outgrowth in motoneurons and regulate neuronal numbers.

## Introduction

G proteins are heterotrimeric proteins that act as intracellular signaling molecules when activated by G protein coupled receptors (GPCR)^1,2^. They are activated when GTP-bound and enter an off-state when bound to GDP^3,4^. It is upon binding of external stimuli that GPCR proteins undergo a conformational change and mediate the activation of the Gα-GDP/Gβγ complex thus acting as guanine nucleotide exchange factor (GEF)^5,6^. The GDP is then released and exchanged by GTP and Gα-GTP and Gβγ are able to signal to downstream effectors^7,8^. This temporal activation will last until the GTP is hydrolyzed either by the intrinsic GTP hydrolysis activity of the Gα subunit or by the activity of regulators of G proteins signaling, RGS, that can dramatically enhance the rate of GTP hydrolysis and act as GTPase activating proteins (GAP)^9–11^. The RGS proteins belong to a large family of Gα GAPs comprising approximately 20 members that interact with the GTP-bound Gα. Thus, through their ability to modulate a myriad of GPCR signaling cascades, RGS proteins are related to numerous human diseases, from cancer to central nervous system disorders, making them attractive drug targets^12–18^.

RGS4 belongs to the R4/B subfamily and the mammalian form is shown to be expressed in developing nervous system and adult brain^19–21^. Several studies point to a role for RGS4 in modulating different neuronal signaling such as dopaminergic, serotonergic, noradrenergic, glutamatergic and opioid^22–24^. *Rgs4* is considered a strong candidate susceptibility gene for schizophrenia and a strong decrease of its mRNA and proteins levels have been described in postmortem samples of schizophrenia patients^25–27^. Xenopus, mice and zebrafish studies have shown abundant expression of *rgs4* in developing nervous system such as neural folds, central and peripheral neuronal precursors^20,28,29^. However, Rgs4 knockout mice showed normal neuronal development^24^ whereas zebrafish morphants also showed normal neuronal development but led to motility defects that could be rescued by activating Akt signaling^28^. Indeed, Rgs4 activity has been linked, mainly through in vitro studies, to several signaling cascades such as the PI3K/Akt, MEK/Erk and the mechanistic regulator of rapamacycin, mTOR with conflicting outcomes^28,30–32^. Therefore, the role for Rgs4 in the development of the nervous system and its link to downstream effectors in vivo remains elusive.

Zebrafish is a great model to study developmental function of genes and to assess the temporal requirements and interactions of signaling cascades in vivo due to its external development and amenability to pharmacological studies. Therefore, we generated a stable zebrafish line with a loss of function mutation in *rgs4* to clarify its role during development. *MZrgs4* mutants showed a sharp decrease in the number of neurons in the central and peripheral nervous system as well as defective motoneurons axon outgrowth and motility defects. Pharmacological and rescue experiments revealed a fundamental role for Rgs4 in regulating neuronal numbers and motoneurons outgrowth that is dependent upon downstream effectors such as PI3K/Akt and MEK/Erk. Most importantly, our results showed a novel role of Rgs4 in the regulation of mTOR activity that is required for nervous system development.

## Results

### Generation of *rgs4* mutant

**Figure 1:**
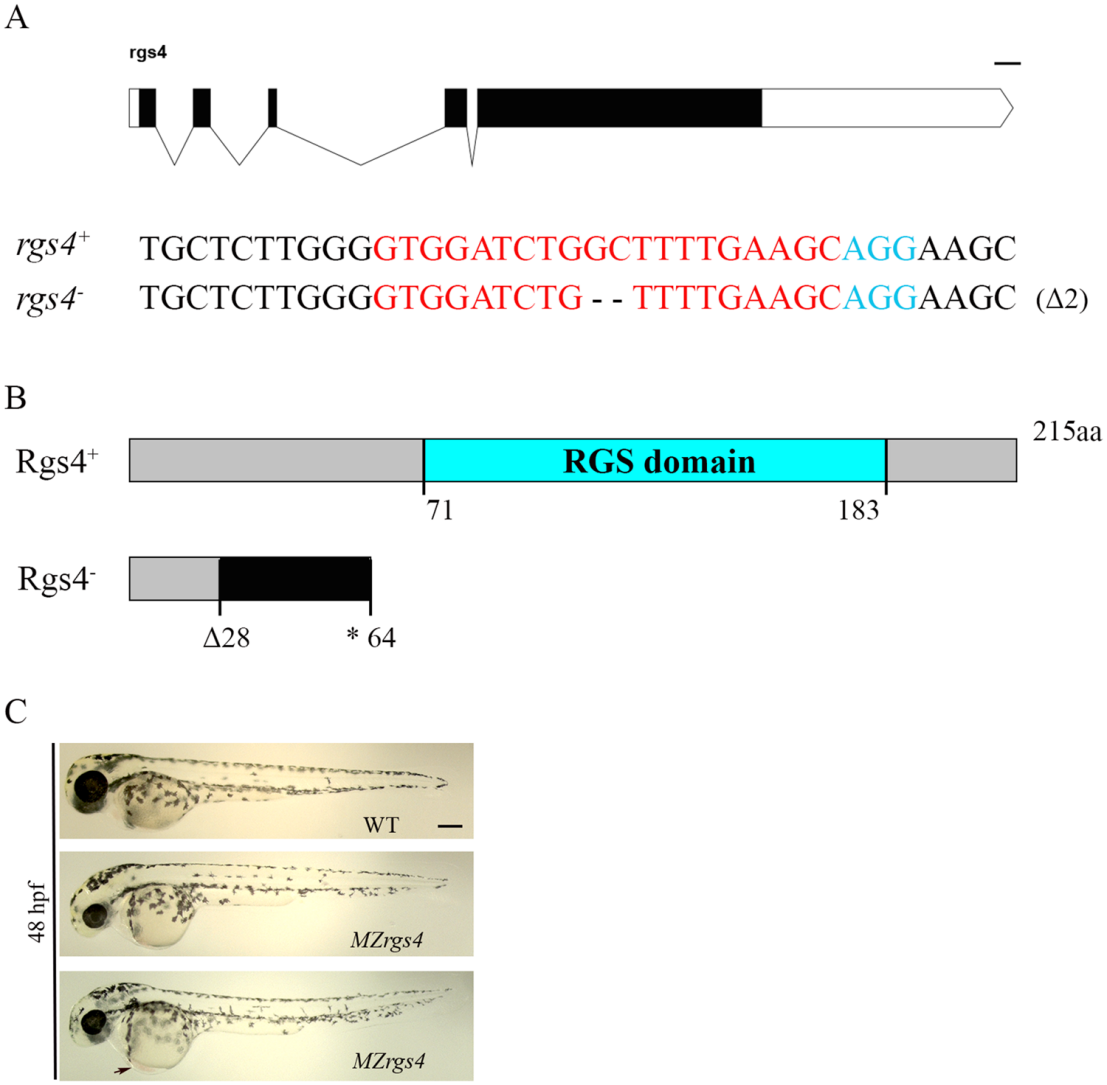
Characterization of *rgs4* mutant. (**A**) Schematic representation of the *rgs4* genomic locus. The extended region on the exon 2 represents the sequence targeted by the CRISPR/Cas9 system. Red: sgRNA binding site. Blue: PAM sequence. *Rgs4*^+^ corresponds to the wild-type allele; *rgs4*^*−*^ is the loss-of-function allele used in this study. Dashes represent the 2 base pairs deletion. (**B**) Schematic of the wild type Rgs4 protein (Rgs4^+^) and the mutated Rgs4 protein (Rgs4^*−*^). The 215 amino acid (aa) long Rgs4^+^ protein contains the RGS domain from amino acid 71 to 183. In *rgs4*^*−/−*^ mutant fish, the 2 base pairs deletion results in a frame shift from the amino acid 28 generating a premature STOP codon at the level of the amino acid 64. (**C**) Lateral views of WT and *MZrgs4* embryos at 48 hpf; black arrow designates a slight heart oedema observed in *MZrgs4* embryos. Scale bar = 200 µm.

It has been shown that *rgs4* mRNA is expressed during early cleavage stages with a ubiquitous expression throughout the whole embryo. Its expression becomes more evident in the nervous system from 12 hpf. Finally, a strong but more restricted expression within clusters of neurons is revealed at later stages^28^, making *rgs4* an attractive candidate to investigate for a role in neural development and function. To assess the role of *rgs4* during development, we generated a *rgs4* mutant using CRISPR/Cas9 technology^33,34^. The introduced mutation engendered a 2 bp deletion within exon 2 that led to a frame shift, a premature stop codon and a significant reduction in *rgs4* expression (Fig. 1A,B; Supplementary Fig. 1). *Rgs4* homozygous mutants (*rgs4*^*−/−*^*)* showed no obvious external defects and were viable to adulthood, which allowed us to analyze maternal-zygotic mutants, *MZrgs4*^*−/−*^ (or *MZrgs4*), that are devoid of all maternal contribution. *MZrgs4* embryos were generally thinner than Wild-Type (WT) embryos with reduced head and eye sizes and slight heart oedema (Fig. 1C, n ≥ 400/500 embryos).

### Rgs4 is required for motoneurons axonal outgrowth and branching

**Figure 2:**
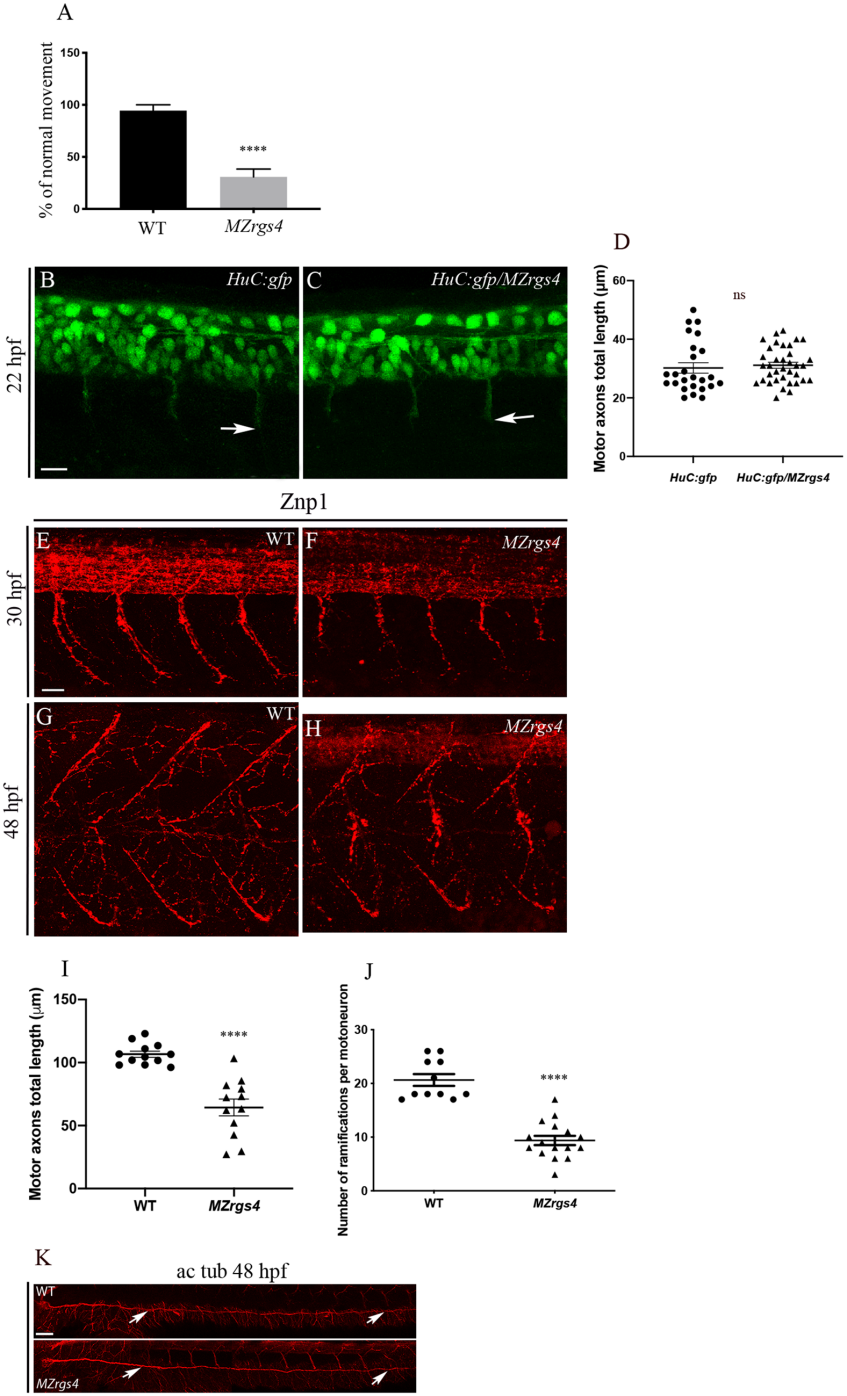
*MZrgs4* embryos show motility and motoneurons axon outgrowth defects. (**A**) Quantification of the percentage of embryos that showed a normal response to the touch test at 48 hpf in WT (average of 94.44 ± 5.556, n = 18) and *MZrgs4* embryos (average of 30.77 ± 7.48, n = 39). (**B**,**C**) Z projections of confocal microscopy images of *HuC:gfp* (**B**) and *HuC:gfp/MZrgs4* mutant (**C**) showing the extending axons of primary motoneurons at 22 hpf. Arrows indicate the tip ends of extending axons. Scale bar = 20 μm. (**D**) Quantification of the motor axons length at 22 hpf in *HuC:gfp* (average of 30.20 ± 1.78 μm, 25 motor axons, n = 9) and *MZrgs4/HuC:gfp* (average of 31.14 ± 1.01 μm, 36 motor axons, n = 12). (**E**–**H**) Z Projections of whole-mount immunostaining for Znp1 showing the extending axons of primary motoneurons at 30 hpf in WT (**E**) and *MZrgs4* (**F**) and the ramifications or branching of these motoneurons at 48 hpf in WT (**G**) and *MZrgs4* embryos (**H**) Scale bar = 25 μm. (**I**) Quantification of the motor axons length at 30 hpf in WT (average of 106.7 ± 2.44 μm, 42 motor axons, n = 12) and *MZrgs4* embryos (average of 64.37 ± 6.66 μm, 50 motor axons, n = 12). (**J**) Quantification of the number of ramifications per motoneuron at 48 hpf in WT (average of 20.64 ± 1.10, 36 motoneurons, n = 11) and *MZrgs4* embryos (average of 9.37 ± 0.86, 53 motoneurons, n = 16). (K) Acetylated tubulin expression in WT and *MZrgs4* embryo at 48 hpf showing the PLLn nerve (white arrows). Scale bar = 50 μm.

A significant percentage of *MZrgs4* showed a defective motility behavior when tested for their touch response and compared to Wild-Type (WT) embryos at 48 hpf, similar to the defect observed in *rgs4* morphants^28^ (Fig. 2A). Since *rgs4* is expressed in the nervous system at this stage and to investigate the underlying motility problem, we first looked at the early development of motoneurons using *Tg(HuC:gfp)* and found no significant difference in the initial development and axonal outgrowth of motoneurons between controls and *MZrgs4* embryos (Fig. 2B–D). We next labeled embryos with the motoneurons marker Znp1 that specifically labels the synaptic protein synaptotagmin in extending axons of the three primary motoneurons, CaP (caudal primary), MiP (middle primary) and RoP (rostral primary) and we focused on CaP neurons that navigate into ventral myotome^35^. We measured the length of the first four outgrowing CaP anterior to the end of yolk extension. The length of developing motoneurons was significantly shorter at 30 hpf in *MZrgs4* embryos when compared to WT (Fig. 2E,F,I). At 48 hpf, the length of developing motor axons was still altered although to a lesser extent, however, the number of ramifications or branching to the muscles was dramatically reduced in *MZrgs4* embryos (Fig. 2G,H,J). To check for the specificity of this axonal defect, we labeled the Posterior Lateral Line nerve (PLLn) that is part of the mechanosensory lateral line system that detects the pattern of water movement in zebrafish^36,37^. *MZrgs4* embryos presented no major defects in PLLn growth at 48 hpf (Fig. 2K).

These results suggest a role for Rgs4 in embryonic motility by regulating the axonal outgrowth and branching of motoneurons similar to what has been proposed following *rgs4* knockdown^28^.

### Rgs4 is required for neuronal development in the PLLg and spinal cord

**Figure 3:**
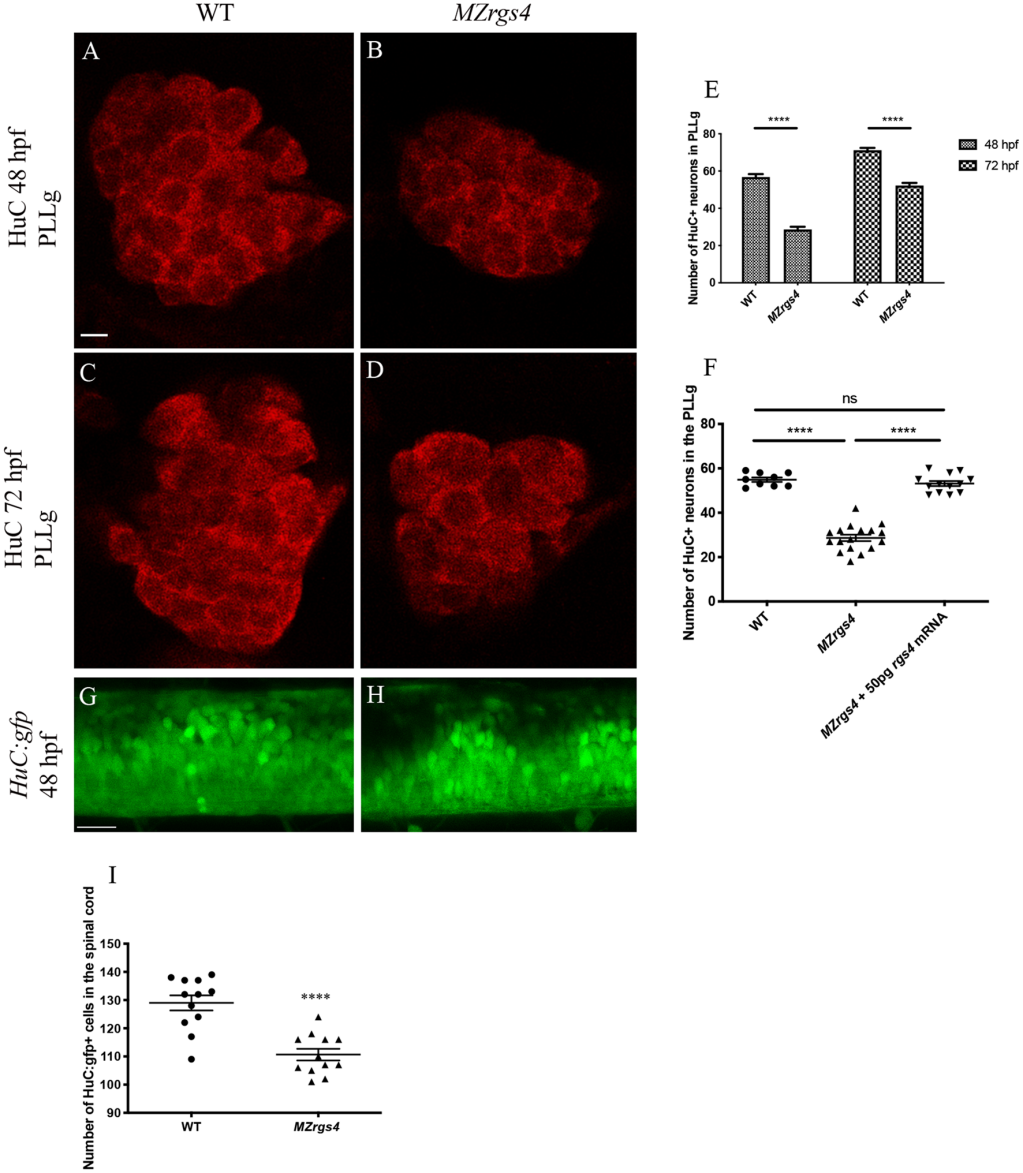
Rgs4 is required for neuronal development in the PLLg and spinal cord. (**A**–**D**), single planes of confocal microscopy images representing the PLLg following HuC labelling at 48 and 72 hpf in WT (A,C) and *MZrgs4* (B,D). Scale bars = 5 μm. (**E**) Graph showing the number of neurons within the PLLg in WT (average of 56.70 ± 1.43, n = 10 at 48 hpf; average of 71.17 ± 1.25, n = 12 at 72 hpf) and *MZrgs4* embryos (average of 29.14 ± 1.71, n = 14 at 48 hpf; average of 52.14 ± 1.33, n = 21 at 72 hpf) at 48 and 72 hpf. (**F**) Quantification of the number of neurons in the PLLg following *rgs4 mRNA* injection in *MZrgs4* mutants at 48 hpf (average of 54.89 ± 1.01 neurons in WT, n = 9; average of 28.65 ± 1.43 neurons in *MZrgs4,* n = 17; average of 53.15 ± 1.13 neurons in *MZrgs4* + *rgs4mRNA*, n = 13). (**G**,**H**) single planes of confocal microscopy images of *Tg(HuC:gfp)* (G) and *Tg(HuC:gfp)/MZrgs4* (H) at 48 hpf. Scale bar = 20 μm. (**I**) Quantification of the number of HuC:gfp + cells within the same area of the spinal cord in WT (average of 129 ± 2.70 gfp + cells, n = 12) and *MZrgs4* (average of 110.7 ± 2.07 gfp + cells, n = 12) at 48 hpf.

*MZrgs4* embryos showed a reduction in head and eye sizes and looked thinner at different stages of development (shown at 48 hpf in Fig. 1). Since *rgs4* is mainly expressed in the nervous system, we investigated whether this phenotype reflects a defective neuronal development. We therefore labeled embryos with the neuronal marker HuC and counted the number of neurons within the PLL ganglia (PLLg) and spinal cord. We observed a significant decrease in the number of neurons in the PLLg of *MZrgs4* embryos at 48 and 72 hpf in comparison to WT (Fig. 3A–E), suggesting a role for *rgs4* in the development of the PLLg. This defect was rescued by injecting 50 pg of *rgs4* mRNA confirming *rgs4* as the gene responsible for the mutant phenotype (Fig. 3F). In addition, a significant decrease in the number of neurons was also observed in the spinal cord of *MZrgs4* embryos (Fig. 3G–I) showing a requirement for Rgs4 in the development of spinal cord.

To further investigate neuronal defects observed in *MZrgs4* embryos, we looked at early determination/differentiation markers that are known to play a major role in neuronal development and assessed their mRNA expression levels at 24 and 48 hpf. *Neurod1* and *neurog1* mRNA expressions were comparable to WT at 24 hpf (Supplementary Fig. 2A). However, when tested at 48 hpf, *neurod1* mRNA expression was significantly decreased in *MZrgs4* embryos while *neurog1* showed no significant difference compared to WT (Supplementary Fig. 2A).

It is also possible that the observed defects are related to a decrease in the survival of newly formed neurons. To test this, we labeled embryos with acridine orange (AO) and looked for cell death specifically within the PLLg and spinal cord. *MZrgs4* embryos showed similar number of AO + cells when compared to WT (Supplementary Fig. 2B–G).

These results point to a role for Rgs4 in regulating neuronal numbers independent of neuronal survival and that *neurod1* mRNA expression is specifically decreased in *MZrgs4* embryos.

### *MZrgs4* embryos show a specific decrease in HuC+ population and an impaired cell proliferation in the central nervous system (CNS)

**Figure 4:**
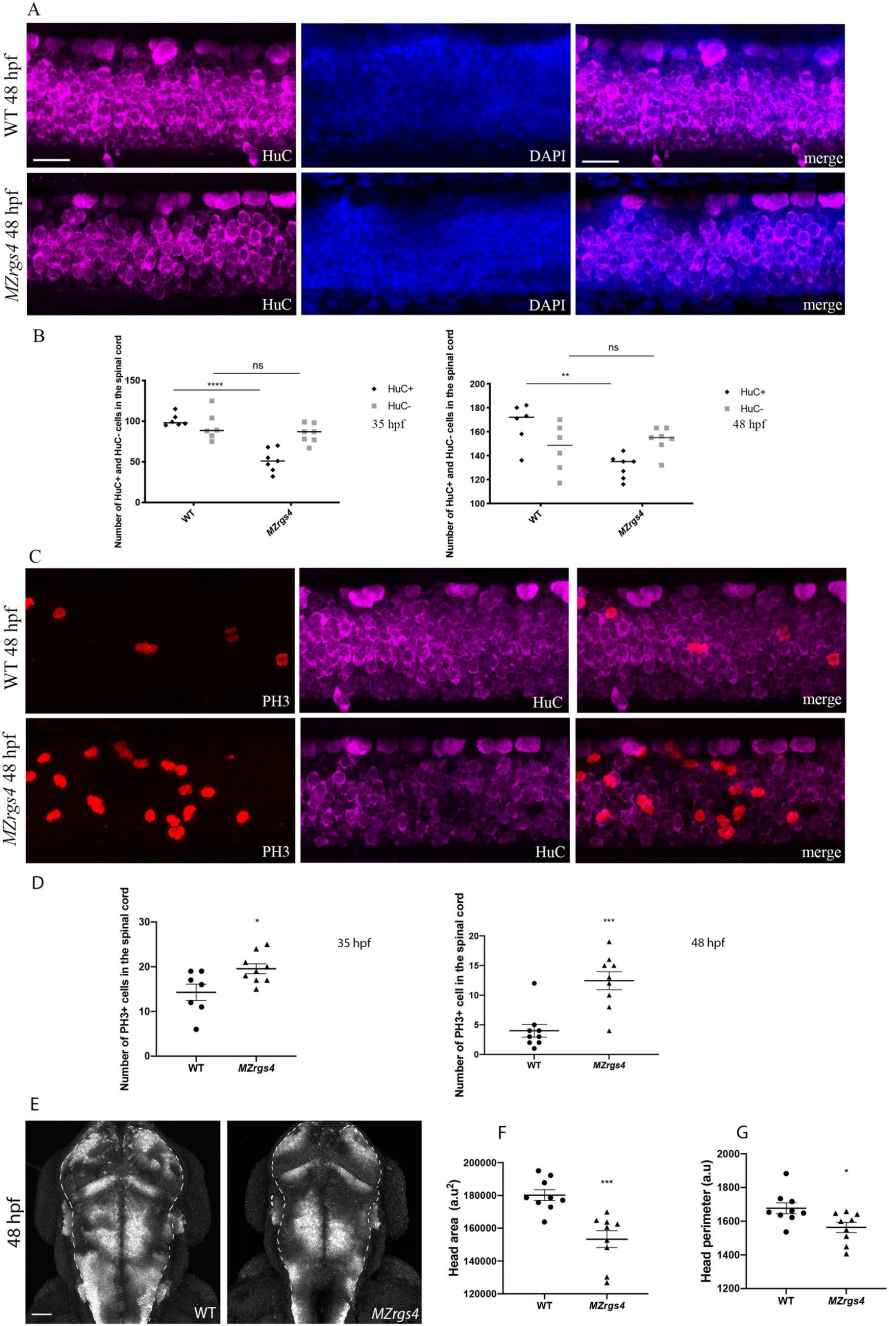
*MZrgs4* embryos show a specific decrease in the number of HuC+ cells and a significant increase in the number of PH3+ cells in the spinal cord. (**A**) Lateral views of spinal cord Z projections (around 15 μm) following whole-mount immunostaining for HuC (left) and counterstained with DAPI (middle) with merged still images (right) in WT (top) and *MZrgs4* larvae (bottom) at 48 hpf. Scale bar = 20 μm. (**B**) Quantification of the number of HuC+ and HuC− cells within the spinal cord in WT (average of 101.2 ± 3.12 for HuC+ ; average of 93.83 ± 7.36 for HuC−, n = 6) and *MZrgs4* larvae (average of 51.86 ± 5.25 for HuC+ ; average of 84.71 ± 4.39 for HuC−, n = 7) at 35 hpf (left) and at 48 hpf in WT (right) (average of 166.7 ± 7.04 for HuC+ ; average of 146.2 ± 8.28 for HuC−, n = 6) and *MZrgs4* larvae (average of 130.3 ± 3.7 for HuC+ ; average of 153.1 ± 3.99 for HuC−, n = 7). (**C**) Lateral views of spinal cord Z projections (around 30 μm for PH3) following whole-mount immunostaining for PH3 (left) and HuC (middle) with merged still images (right) in WT (top) and *MZrgs4* larvae (bottom) at 48 hpf. (**D**) Quantification of the number of PH3+ cells within the spinal cord in WT (average of 14.29 ± 1.82 at 35 hpf, n = 7; average of 4 ± 1.08 at 48 hpf, n = 9) and *MZrgs4* larvae (average of 19.56 ± 1.10 at 35 hpf, n = 9; average of 12.44 ± 1.51 at 48 hpf, n = 9). (**E**) Dorsal views of head Z projections (around 400 μm) following HuC whole-mount immunostaining in WT and *MZrgs4* larvae at 48 hpf. Dashed lines represent the nervous system. Scale bar = 50 μm. (**F**) Quantification of the head area in WT (average of 180,194 ± 3289, n = 9) and *MZrgs4* larvae (average of 153,316 ± 5151, n = 9) at 48 hpf. (**G**) Quantification of the head perimeter in WT (average of 1677 ± 31.90, n = 9) and *MZrgs4* larvae (average of 1564 ± 30.24, n = 9) at 48 hpf.

How does Rgs4 control CNS development? To start answering this question, we specifically labeled embryos with neuronal marker HuC along with the M-phase mitotic marker phospho-histone 3 (PH3) and we counterstained cell nuclei with DAPI to outline neural tissue. Z stacks were captured from a lateral view, allowing to visualize the whole tissue within the spinal cord and count the number of HuC+ and HuC− cell populations as well as PH3+ cells. From these stacks, we observed that *MZrgs4* embryos show a specific and significant decrease in the number of HuC+ cells while HuC− cells numbers were comparable to controls (Fig. 4A,B). Moreover, these data showed a significant increase in the number of PH3+ cells in *MZrgs4* embryos pointing to an imbalance of proliferation/differentiation rates that impairs the number of HuC+ cells within the spinal cord (Fig. 4C,D). Finally, we captured z stacks from dorsal views and we confirmed the decrease in the perimeter and area of *MZrgs4* larvae heads when compared to WT (Fig. 4E-G).

These data strongly suggest a specific requirement for Rgs4 in the neuronal development of the CNS, and that the critical balance of proliferation versus differentiation that generates the correct number of neurons is impaired in *MZrgs4* mutants.

### *MZrgs4* embryos show a decreased Akt signaling activity

**Figure 5:**
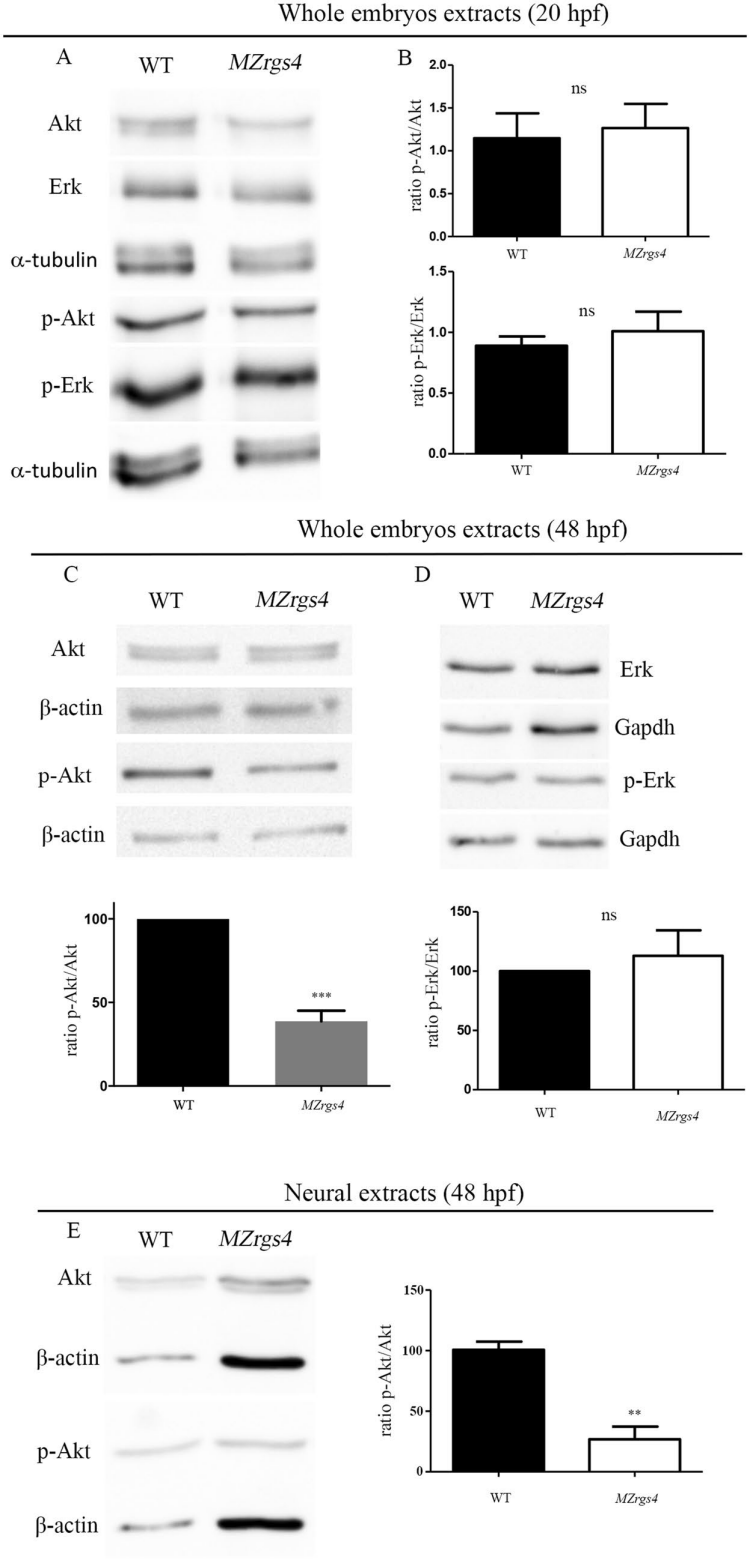
Loss of *rgs4* function alters Akt signaling in neural cells. (**A**,**B**) Immunoblotting of lysates from zebrafish embryos at 20 hpf. Akt and p-Akt amounts were normalized to α-tubulin and the ratio of p-Akt relative to Akt was compared between WT and *MZrgs4* lysates. No significant difference was observed between the two groups. Erk and p-Erk amounts were normalized to α-tubulin and the ratio of p-Erk relative to Erk was compared between WT and *MZrgs4* lysates. No significant difference was observed between the two groups. (**C**) Immunoblotting of lysates from zebrafish embryos at 48 hpf. Akt and p-Akt amounts were normalized to β-actin and the ratio of p-Akt relative to Akt was compared between WT and *MZrgs4* lysates. *MZrgs4* embryos showed a significant decrease in the amount of p-Akt/Akt in comparison to WT. (**D**) Immunoblotting of lysates from zebrafish embryos at 48 hpf. Erk and p-Erk amounts were normalized to Gapdh and the ratio of p-Erk relative to Erk was compared between WT and *MZrgs4* lysates. No significant difference was observed between the two groups. (**E**) Immunoblotting of neural lysates (or extracts) from zebrafish embryos at 48 hpf. Akt and p-Akt amounts were normalized to β-actin and the ratio of p-Akt relative to Akt was compared between WT and *MZrgs4* lysates. *MZrgs4* embryos showed a significant decrease in the amount of p-Akt/Akt in comparison to WT.

Rgs4 is a multitask protein that interacts with several receptors and can modulate a plethora of cellular signaling with different outcomes that is most likely to be dependent on the cellular and temporal context of its activity^12^. We therefore asked how does Rgs4 activity regulate both axonal outgrowth and neuronal development in vivo and what are the signaling pathways linked to its function? We first tested whether PI3K/Akt and MEK/Erk pathways are altered in *MZrgs4* embryos. To do so, we quantified the expression levels of the active phosphorylated form of Akt (p-Akt) relative to the expression of Akt at different timepoints, including 20 hpf and 48 hpf. This ratio was comparable between WT and *MZrgs4* embryos at 20 hpf but significantly decreased in *MZrgs4* embryos at 48 hpf in comparison to WT (Fig. 5A-C; Supplementary Figs. 3–5). When testing for Erk activity in *MZrgs4* embryos, we detected no change in the expression levels of phosphorylated Erk relative to total Erk at 20 and 48 hpf after analysis of whole embryos extracts (Fig. 5A,B,D; Supplementary Figs. 3–5). We next wondered whether Rgs4 is required for Akt activity specifically within the nervous system, we therefore dissociated embryos and generated neural cells enriched cultures^38^ from WT and *MZrgs4* embryos at 48 hpf to quantify p-Akt/Akt ratio in primary neural cultures. The latter was significantly decreased in *MZrgs4* neural extracts in comparison to WT (Fig. 5E; Supplementary Figs. 3–5).

This result suggests that Akt pathway activity is impaired in the absence of Rgs4 and that the latter positively regulates Akt signaling pathway during development.

### Rgs4 motoneurons branching and neuronal development activities are mediated through Akt and Erk activation

**Figure 6:**
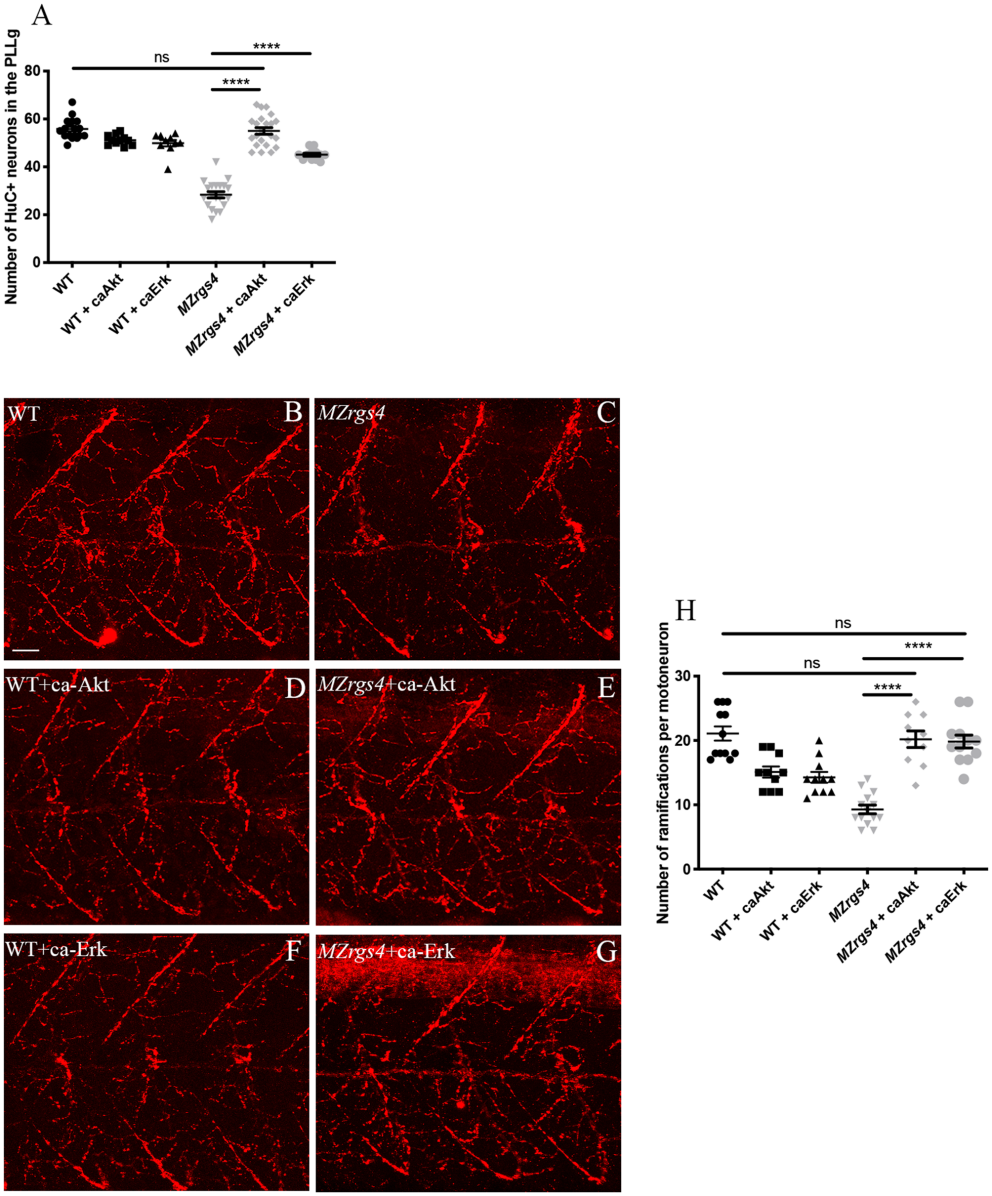
Akt and Erk activities mediate Rgs4-dependent PLLg development and motoneurons branching. (**A**) Quantification of the number of neurons in the PLLg in WT (average of 55.83 ± 1.02, n = 18), WT + caAkt (average of 51.18 ± 0.65, n = 11), WT + caErk (average of 49.90 ± 1.35, n = 10), *MZrgs4* (average of 28.35 ± 1.28, n = 20), *MZrgs4* + caAkt (average of 55 ± 1.36, n = 22) and *MZrgs4* + caErk (average of 45.08 ± 0.64, n = 12). (**B**–**G**) Z projections of whole-mount immunostaining for Znp1 labeling the ramifications of motoneurons in WT (**B**), *MZrgs4* (**C**) WT + caAkt (**D**), *MZrgs4* + caAkt (**E**), WT + caErk (**F**) and *MZrgs4* + caErk (**G**) at 48 hpf. Scale bar = 20 μm. (**H**) Quantification of the number of ramifications per motoneuron in WT (average of 21.08 ± 1.10, 40 motoneurons, n = 12), WT + caAkt (average of 15.10 ± 0.87, 30 motoneurons, n = 10), WT + caErk (average of 14.27 ± 0.83, 33 motoneurons, n = 11), *MZrgs4* (average of 9.28 ± 0.66, 46 motoneurons, n = 14), *MZrgs4* + caAkt (average of 20.20 ± 1.28, 32 motoneurons, n = 10) and *MZrgs4* + caErk (average of 19.83 ± 1.00, 40 motoneurons, n = 12) at 48 hpf.

Our results showed that Akt signaling activity is significantly decreased in *MZrgs4* mutants, it is also possible that Erk activity is dysregulated in this case but given the dynamic nature of GAP activity we might have not been able to detect it. To assess whether Rgs4 activity is mediated by PI3K/Akt and/or MEK/Erk pathways in vivo, we first took advantage of two pharmacological compounds, LY294002 and U0126, that specifically and selectively alter the activity of these two pathways respectively^39–42^ and checked whether they are required for the development of PLLg and motoneurons branching. We treated embryos with each of the two compounds separately (LY294002; U0126) or in combination (LY294002 + U0126) from 24 till 48 hpf and counted the number of HuC+ neurons in the PLLg. Our results showed a sharp decrease in the number of neurons in the PLLg in all treated groups in comparison to untreated WT (Supplementary Fig. 6A), suggesting that the activity of these pathways is required for neuronal development of PLLg. The next step was to test whether Rgs4 activity is mediated by these pathways. To do so, we injected constitutive active form of Akt (caAkt)^28^ and Erk that we generated (caErk) (see materials and methods) in *MZrgs4* and WT embryos. Injection of caAkt into *MZrgs4* embryos fully restored neuronal numbers in these embryos, while injection of caErk partially rescued the phenotype (Fig. 6A). Along the same lines, we tested for a possible role for these pathways in motoneurons branching following the same strategy as for PLLg analysis. Our results showed a requirement for these pathways in motoneurons branching with a possible redundancy between the two (Supplementary Fig. 6B–E). Moreover, injection of caAkt or caErk fully rescued the motoneurons branching defects observed in *MZrgs4* embryos (Fig. 6B–H).

Overall, these results indicate a requirement for the two signaling pathways PI3K/Akt and MEK/Erk in neuronal development and motoneurons branching and that Rgs4 activity is mediated by these pathways.

### Rgs4 is required for mTOR activity

**Figure 7:**
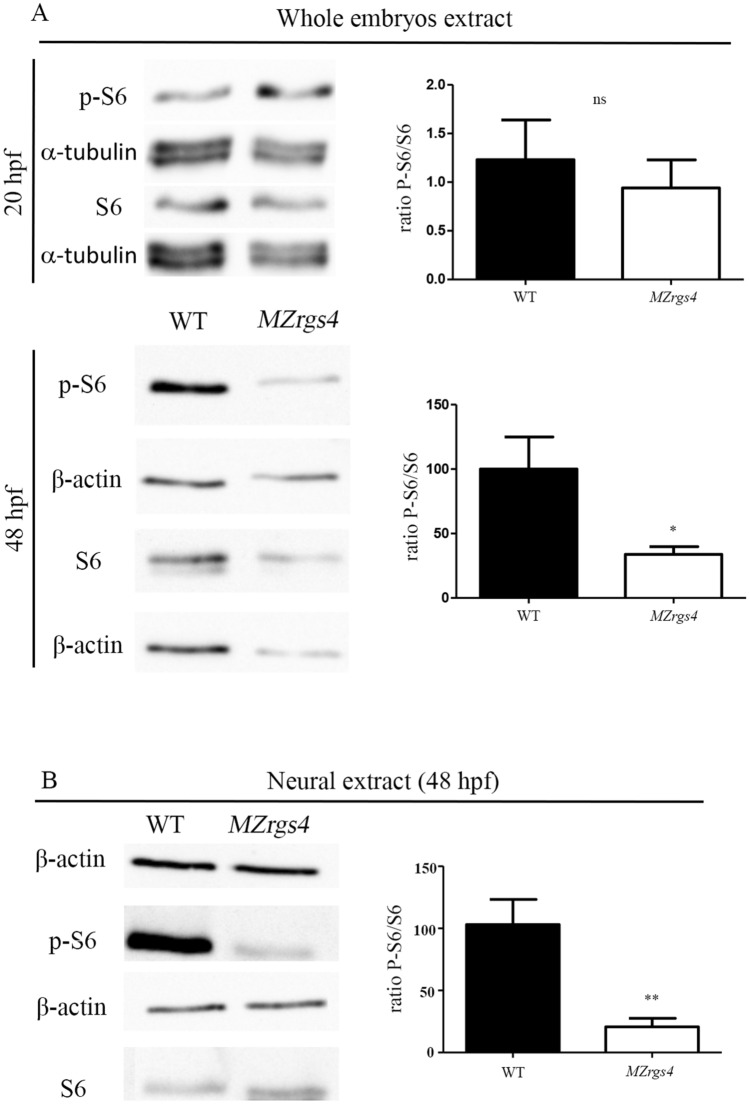
Loss of *rgs4* function alters mTOR signaling in neural cells (**A**) Immunoblotting of lysates from zebrafish embryos at 20 hpf. S6 and p-S6 amounts were normalized to α-tubulin and the ratio of p-S6 relative to S6 was compared between WT and *MZrgs4* lysates. No significant difference was observed between the two groups. Immunoblotting of lysates from zebrafish embryos at 48 hpf. S6 and p-S6 amounts were normalized to β-actin and the ratio of p-S6 relative to S6 was compared between WT and *MZrgs4* lysates. *MZrgs4* embryos show a significant decrease in the amount of p-S6/S6 in comparison to WT. (**B**) Immunoblotting of neural lysates from zebrafish embryos at 48 hpf. S6 and p-S6 amounts were normalized to β-actin and the ratio of p-S6 relative to S6 was compared between WT and *MZrgs4* lysates. *MZrgs4* embryos showed a significant decrease in the amount of p-S6/S6 in comparison to WT.

A previous study identified Rgs4 as a novel target of mTOR inhibition in a mouse glioma model^31^ and since the PI3K/Akt that mediates Rgs4 activity is one of the major drivers of mTOR activity, we wondered whether Rgs4 is required for mTOR activation in vivo. We first tested the activity of mTOR downstream effector, S6, in *MZrgs4* embryos by western blot analysis. Extracts from whole embryos were analyzed for the expression levels of the phosphorylated form of S6 (p-S6) relative to the expression of S6 at 20 and 48 hpf. This ratio was comparable between WT and *MZrgs4* embryos at 20 hpf; however, a significant reduction in *MZrgs4* embryos compared to WT was observed at 48 hpf (Fig. 7A; Supplementary Figs. 3–5). This result stimulated our interest in investigating whether mTOR activity is required for neuronal development and motoneurons branching and to test a possible link between Rgs4 and mTOR pathway. For this, we treated embryos with rapamycin, a commonly used compound that specifically inhibits mTOR, between 24 and 48 hpf. Treated WT embryos showed a significant decrease in the number of HuC+ neurons in the PLLg (Supplementary Fig. 7A), reflecting a role for mTOR in the development of PLLg. If Rgs4 activity in neuronal development is mediated by mTOR, then activating mTOR in *MZrgs4* embryos should rescue this phenotype. We therefore tested a new mTOR activator compound (MHY 1485) and treated *MZrgs4* embryos with MHY from 24 till 48 hpf^43,44^. *MZrgs4* treated embryos showed a significant increase in the number of HuC+ neurons in PLLg (Supplementary Fig. 7A), reflecting a link between Rgs4 and mTOR activity. We also analyzed the role of mTOR and the link between Rgs4 and mTOR activity in motoneurons branching using the same strategy as for PLLg analysis. Our results suggested a significant role for mTOR in motoneurons branching and that Rgs4 activity is mediated, at least partially, by mTOR activity (Supplementary Fig. 7B–F). To test whether Rgs4 positively regulates mTOR signaling within the nervous system, we generated neural cells enriched cultures from WT and *MZrgs4* embryos at 48 hpf, and mTOR activity was assessed by western blot analysis similar to whole embryos extracts. Results showed that the ratio of phosphorylated S6 relative to S6 is significantly down-regulated in neural cells extracts of *MZrgs4* embryos (Fig. 7B; Supplementary Figs. 3–5).

These results showed an impaired mTOR activity in the absence of Rgs4 and that the latter is a positive regulator of mTOR.

### PI3K/Akt and MEK/Erk activities are linked to mTOR signaling in *MZrgs4* embryos

Results so far suggested that neuronal Rgs4 activity is mediated through different signaling pathways, that are PI3K/Akt, MEK/Erk and mTOR. Given the established PI3K/Akt/mTOR signaling^45^ and the crosstalk between MEK/Erk and mTOR pathways^46,47^, we asked whether Akt and Erk activities that relay Rgs4 to downstream effectors are linked to mTOR activity. We reasoned that if Akt and Erk activities are mediated through mTOR, then activating Akt or Erk in *MZrgs4* and treating embryos with rapamycin to inhibit mTOR activity would impair Akt and Erk rescuing ability. Indeed, embryos injected with caAkt or caErk and treated with rapamycin showed a significant decrease in the number of neurons in PLLg in comparison with untreated *MZrgs4* embryos injected with caAkt or caErk (Supplementary Fig. 7G,H).

This result shows that Akt and Erk activities are, at least partially, dependent upon mTOR signaling to regulate neuronal development.

We followed the same strategy to analyze signaling interactions required for motoneurons branching in *MZrgs4* embryos. As observed for PLLg development, embryos injected with caAkt or caErk and treated with rapamycin showed a sharp decrease in the number of ramifications along motoneurons in comparison with *MZrgs4* injected embryos. However, in this case, the injected/treated embryos were comparable to *MZrgs4* suggesting that Rgs4 function in motoneurons branching mediated by PI3K/Akt and MEK/Erk is strongly linked to mTOR signaling (Supplementary Fig. 7I–N).

### Rgs4 function is required within motoneurons and is mediated autonomously by Akt and Erk activation to drive motoneurons axonal outgrowth

**Figure 8:**
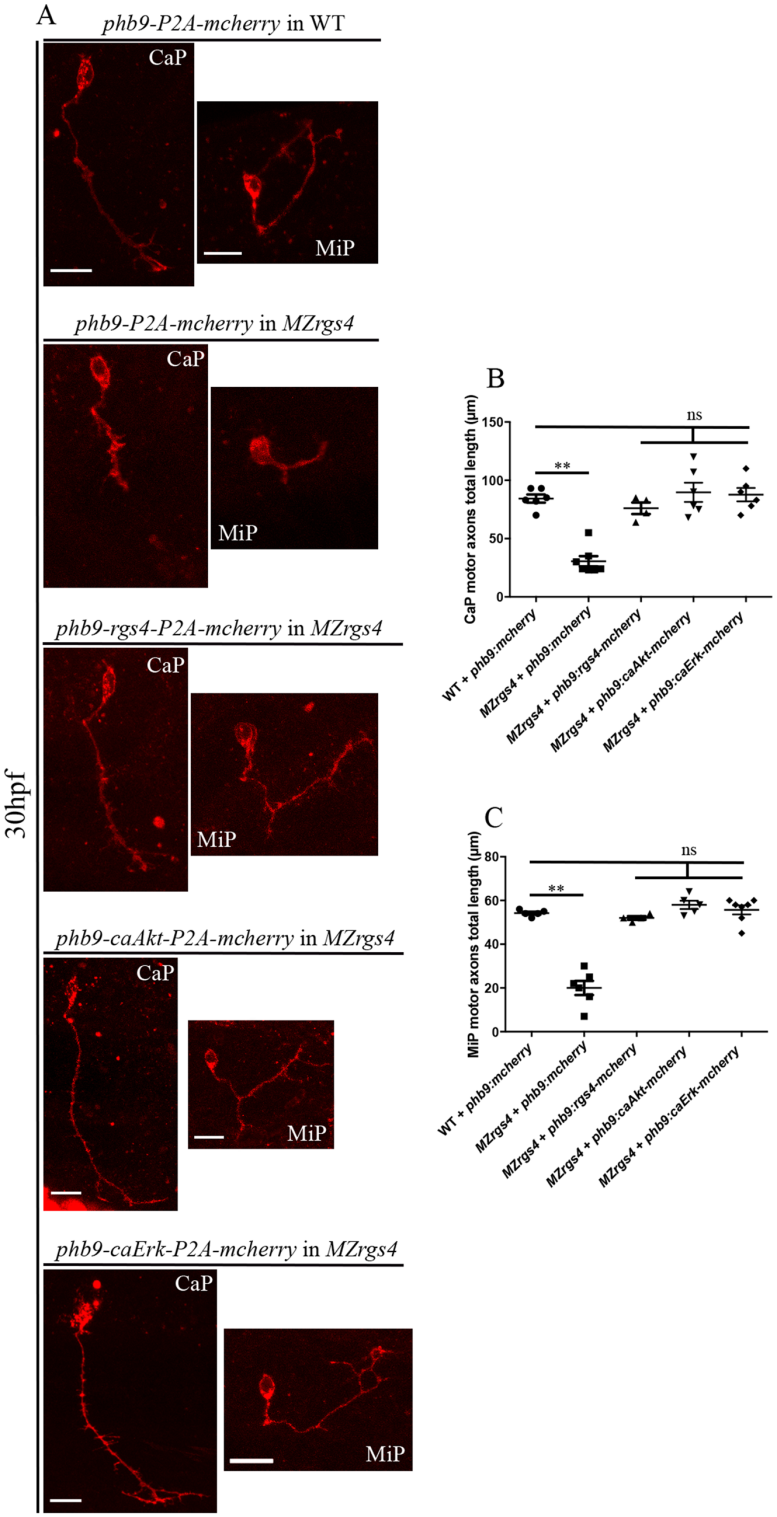
Rgs4 function, Akt and Erk activities that mediate Rgs4-dependent motoneurons outgrowth are all required within motoneurons. (**A**) Representative maximum projections of z-stack optical sections of CaP and MiP at 30 hpf following injection of different constructs allowing the expression of mcherry in WT and *MZrgs*4, Rgs4-P2A-mcherry in *MZrgs4*, caAkt-P2A-mcherry in *MZrgs4* and caErk-P2A-mcherry in *MZrgs4* specifically in motoneurons under the control of hb9 promoter. (**B**) Quantification of individual CaP motor axons length following injection of *hb9:mcherry* in WT (average of 84.33 ± 3.48 μm, 6 CaP, n = 4) and *MZrgs4* (30.57 ± 4.41 μm, 7 CaP, n = 5), injection of *hb9:rgs4-P2A-mcherry* in *MZrgs4* (average of 76 ± 4.91 μm, 4 CaP, n = 4), injection of *hb9:caAkt-P2A-mcherry* in *MZrgs4* (average of 89.67 ± 8.24 μm, 6 CaP, n = 6) and injection of *hb9:caErk-P2A-mcherry* in *MZrgs4* (average of 87.67 ± 5.73 μm, 6 CaP, n = 6). (**C**) Quantification of individual MiP motor axons length following injection of *hb9:mcherry* in WT (average of 54.20 ± 0.66 μm, 5 MiP, n = 4) and *MZrgs4* (20 ± 3.23 μm, 6 MiP, n = 5), injection of *hb9:rgs4-P2A-mcherry* in *MZrgs4* (average of 52 ± 0.82 μm, 4 MiP, n = 4), injection of *hb9:caAkt-P2A-mcherry* in *MZrgs4* (average of 58 ± 1.92 μm, 5 MiP, n = 5) and injection of *hb9:caErk-P2A-mcherry* in *MZrgs4* (average of 55.71 ± 2.05 μm, 7 MiP, n = 7). ns, non significant.

Motoneurons axonal pathfinding and growth are dependent on attractive and repulsive stimuli generated from nervous and non-nervous surrounding tissues and from intrinsic motoneurons signaling^48–53^, so it was important to establish whether Rgs4 is required within motoneurons to drive their early outgrowth. To test this, we forced the expression of Rgs4 in *MZrgs4* embryos specifically in motoneurons under the control of *hb9* promoter. *MZrgs4* mutant embryos were injected with p*hb9:rgs4-2A-mcherry-CaaX* or p*hb9-2A-mcherry-CaaX* at one cell stage and assessed at 30 hpf for mCherry expression in individual motoneurons along the spinal cord. We identified two major groups of motoneurons following injections, the CaP and MiP and we measured the length of their axonal growth. *MZrgs4* embryos injected with p*hb9:rgs4-2A-mcherry-CaaX* showed normal axonal growth and presented no significant difference to WT embryos injected with p*hb9-2A-mcherry-CaaX* (Fig. 8A-C), while *MZrgs4* embryos injected with p*hb9-2A-mcherry-CaaX* had significantly shorter axons than the other two groups. To assess whether Rgs4 activity is mediated through Akt and Erk activity within motoneurons, we forced the expression of caAkt and caErk specifically within motoneurons in *MZrgs4* embryos under the control of *hb9* promoter. *MZrgs4* embryos injected with p*hb9:caAkt-2A-mcherry-CaaX* or p*hb9:caErk-2A-mcherry-CaaX* showed normal axonal growth as observed in WT embryos (Fig. 8A–C).

This result strongly suggests a requirement for Rgs4 within motoneurons to drive their axonal outgrowth and that its activity is mediated through Akt and Erk in motoneurons.

## Discussion

The G proteins are known to control several fundamental signal-transduction pathways with major clinical significance^12^. Hence, targeting the activity of these proteins is subject to intensive pharmaceutical studies mainly focusing on GPCRs that relate extracellular signaling to G protein activation. RGS have emerged as new components that finely tune the activity of GPCRs and present themselves today as potential drug targets aiming to enhance or attenuate GPCR signaling^17^. One particular member of this family is RGS4 that is mainly expressed in the nervous system and has been linked to several neuronal pathways but there are conflicting results on how RGS4 regulates neuronal signaling and what are the downstream effectors that mediate its function^12^. In this article, we generated a stable *MZrgs4*^*−/−*^ zebrafish mutant and provided evidence that *rgs4* is required for neuronal development in the central and peripheral nervous system. Moreover, we showed that Rgs4 is required within motoneurons for axonal outgrowth and branching.

How does Rgs4 regulate neuronal development? Many GPCR and RGS are enriched in the nervous system and some, such as RGS3 and GPRC5B contribute to mammalian neurogenesis^54,55^. Rgs4 is known to exhibit a very dynamic neural expression during mouse embryonic development that largely matches the neural type determinant *phox2b* expression^20^; it is also highly enriched in neural progenitors in zebrafish embryos^28^, however there is no in vivo evidence so far that links Rgs4 activity to neuronal development. Moreover, the molecular mechanisms that connect Rgs4 to neuronal development are not known. We hypothesized that Rgs4 enables signal transduction that promotes neuronal growth and we focused on PI3K/Akt and MEK/Erk pathways. First, using the neuronal differentiation marker HuC, we observed a significant reduction in the number of differentiated neurons in the spinal cord and PLLg of *MZrgs4* embryos that coincides with a significant decrease in *neurod1* mRNA expression, independent of neuronal survival. The fact that *neurog1* and early *neurod1* mRNA expressions in *MZrgs4* embryos were comparable to WT suggests a role for Rgs4 in terminal neuronal differentiation. Moreover, spinal cord analysis showed a specific decrease in the number of HuC( +) cells and an increase in the number of PH3( +) cells, pointing to an incorrect balance of proliferation and differentiation within the nervous system that selectively impairs neuronal numbers. It is possible that a high percentage of these proliferating cells in *rgs4*^*−/−*^ larvae would struggle to exit mitosis and thus contribute to the significant decrease in the number of differentiated neurons and reduced head size. The accumulation of mitotic cells in the CNS leading to microcephaly has indeed been reported in several zebrafish mutants and is a common feature of the autosomal recessive primary microcephaly (MCPH) condition^56,57^. Further time-lapse clonal analysis is required to test this hypothesis in *rgs4* mutants. Second, using a molecular readout of Akt activity, we found that Akt activity was significantly reduced in *MZrgs4* mutant zebrafish larvae and more specifically in *MZrgs4* neural extracts. Third, we established an important role for Akt signaling in neuronal development and we followed up this observation to show here that forcing the expression of Akt fully restores the number of neurons in *MZrgs4* mutants. Whereas *MZrgs4* mutant zebrafish larvae showed a decrease in Akt signaling, we observed no changes in Erk activity. One interpretation of this result is the very dynamic expression of Rgs4 that may have prevented the detection of a significant change in the activity of this pathway since forcing the expression of Erk was also sufficient to increase the number of neurons in *MZrgs4* mutant larvae, although to a lesser extent than Akt. Together, our data indicate that Akt and Erk activities are both required for neural development, they mediate Rgs4 dependent neuronal development, but that Rgs4 mainly signals through Akt to promote this process. It is important to note that Rgs4 is a positive regulator of Akt activity during development. Another interesting observation is the requirement of Rgs4 in regulating neuronal numbers in zebrafish while its activity is dispensable for mice neurogenesis^24^. This raises the possibility of a significant redundancy with other RGS in mice that compensates the lack of RGS4. Our current work also provides insights into the downstream signaling of PI3k/Akt and MEK/Erk pathways linking Rgs4 activity to neuronal development. While other studies described a role for Akt in mediating Rgs4 function^28,30,32^ however, little is yet known about other signaling pathways that help achieve this role. In the current work, we found that inhibiting mTOR signaling with rapamycin decreased the number of neurons within the PLLg showing that mTOR activity is required for its development. Moreover, we also found that activating Akt or Erk signaling while inhibiting mTOR activity failed to restore neuronal numbers in *MZrgs4* mutant larvae. Together, our data indicate that Rgs4 mediates neuronal development via Akt, Erk and mTOR signaling and that mTOR activity is specifically reduced within *MZrgs4* neural cells. Nevertheless, considerably more work will be required to understand the functional interaction between Rgs4, these signaling pathways and others as there is still much to learn about how they regulate neurogenesis and nervous system growth.

Because *rgs4* is highly expressed in the spinal cord and *MZrgs4* embryos show motility defects, we hypothesized that Rgs4 enables signaling transduction that promote motoneurons outgrowth. A previous study has shown a role for Akt signaling in mediating Rgs4-dependent function in motoneurons branching following *rgs4* knockdown^28^, however, several aspects of *rgs4* function in this process remained unresolved. For instance, can Erk signaling also mediate this function? Is Rgs4 required within motoneurons for their outgrowth? Is there a link between the Rgs4/Akt and Rgs4/Erk pathways? What are the downstream effectors?

Our study provides novel insights into the mechanisms of motoneurons outgrowth in vivo. We presented several lines of evidence that the requirement for Rgs4 in motoneurons outgrowth reflects, Rgs4 dependent PI3K/Akt/mTOR and MEK/Erk/mTOR signaling pathways. We found that Akt, Erk and mTOR activities are all required for motoneurons outgrowth and that injection of caAkt or caErk rescues motoneurons outgrowth defects in *MZrgs4* mutant larvae and importantly, this rescue is impaired following rapamycin treatment. Moreover, our data showed a requirement for Rgs4 within motoneurons to drive their outgrowth. Accordingly, we found that motoneuron-specific expression of a constitutively active form of Akt or Erk rescued motoneurons outgrowth defects in *MZrgs4* embryos. It is important to note that we could only detect a significant reduction in Akt and mTOR activities in *MZrgs4* larvae or neural extracts at 48 hpf while we provide evidence of an earlier role for Rgs4 upstream of Akt and Erk during motoneurons outgrowth. It is possible that a certain threshold of Akt, Erk or mTOR inactivity has to be reached for a significant change to be detected by Western blot that does not necessarily reflects their very dynamic activity throughout development.

Together, our work showed an important role for Rgs4 in neuronal development and motoneurons outgrowth by regulating the activity of at least two fundamental signaling pathways, PI3k/Akt, MEK/Erk. Most importantly, we discovered that mTOR activity is specifically impaired within the nervous system in the absence of Rgs4 and that neuronal defects observed in *MZrgs4* mutants can be rescued, at least partially, by enhancing mTOR activity. Because Rgs4, Akt and mTOR are all linked to several human diseases such as schizophrenia and Parkinson^25–27,58^ and given the new insights that this work provided strongly associating the three in neurodevelopment and motoneurons outgrowth, it is possible that similar defects intervene in schizophrenia and Parkinson Disease. We here provide a novel model for study of *rgs4* function in vertebrates and for dissecting neuronal Rgs4-dependent mechanisms in physiological and pathological conditions.

## Methods

All methods were carried out in accordance with relevant guidelines and regulations.

*Embryo care* Embryos were staged and cared for according to standard protocols (https://zfin.org/zf_info/zfbook/cont.html). F0 injected animals were outcrossed with WT to check for germline transmission. F1 *rgs4*^*−/−*^ mutants were generated by crossing heterozygous *rgs4*^+*/−*^ animals. Maternal zygotic (*MZrgs4*^*−/−*^* or MZrgs4*) animals were generated by crossing homozygous *rgs4*^*−*/*−*^ adult fish. Experiments were conducted throughout different generations of *rgs4*^*−/−*^ mutants during which the *rgs4*^*−/−*^ mutants were outcrossed at least twice with WT. *Tg(huc:gfp)* stable transgenic line, that label neurons was used in this study^59^. All animal experiments were conducted in compliance with the ARRIVE guidelines and with approved protocols at Inserm by DDPP Val de Marne, France under license number F 94–043-013.

### Rgs4 CRISPR mutagenesis

#### sgRNA generation

sgRNA guide sequence ‘GTGGATCTGGCTTTTGAAGC’, targeting *rgs4* exon 2 was designed using CRISPOR program (www.crispor.tefor.net). The sgRNA was generated using a cloning free method^60^. In vitro transcription of the sgRNA was performed using the HiScribe T7 Quick High yield RNA synthesis kit (New England Biolabs) and sgRNA was purified using ZYMO Clean&Concentrator-5 kit (Ozyme).

#### Injections and mutant carrier identification

To induce targeted mutagenesis at the *rgs4* locus, 300 ng/µl of sgRNA was injected into one-cell stage zebrafish embryos together with Cas9 endonuclease (NEB M0386M; final concentration: 20 μM). Pools of embryos were digested to extract genomic DNA (to perform PCR using the locus-specific forward and reverse primer set, followed by DNA sequencing experiments). Injected embryos were grown to adulthood and screened for mutation in their offspring. A mutant carrier that showed a deletion of 2 nucleotides was used in this study (See Fig. 1). Mutants showed a significant decrease in *rgs4* expression, but we could not detect protein levels of expression due to a lack of an antibody that specifically detects zebrafish Rgs4.

To discriminate mutants from WT, a locus specific PCR was performed using DreamTaq enzyme (Fisher scientific):

Forward rgs4: 5’TGTTGACCACAGTGTCCTTCA3’ and,

Reverse rgs4: 5’ACACTCTGGGGAAAGAGCAA-3.

The PCR Program is as follows:

**Table:** 

Denaturation	98°	30 s
PCR 35x	98	10 s
	65°	20 s
	72°	20 s
Final Extension	72°	5 min
Hold	4°	infini

PCR product was digested with MwoI enzyme (NEB) and seen on 2% agarose gels. The digestion profiles are WT: 236 bp + 21 bp + 91 bp; *rgs4*^+*/−*^*:* 236 bp & 345 bp; *rgs4*^*−/−*^ 345 bp.

### Plasmids constructs

The Rgs4-P2A-mCherry cassette allowing simultaneous expression of Rgs4 and membrane localized mCherry separated by the self-cleaving P2A peptide was generated by PCR amplification. The 5’- TTTGCAACTATGTGTAAAGGGCTTGCTGCTCTTC -3’ forward and 5’- GAGAGAAGTTCGTGGCTCCGGATCCGGCATAACTAGGCAAACACTGACTG -3’ reverse primers were used onto the pCS2-Rgs4 plasmid (a gift from Chia-Jung Shen) and the 5’- CAGTCAGTGTTTGCCTAGTTATGCCGGATCCGGAGCCACGAACTTCTCTC -3’ forward and 5’-CTATGACCATGATTACGCCAAG-3’ reverse primers onto the pCS2-mCherry-CAAX plasmid.

The caAkt-P2A-mCherry cassette was also generated by PCR amplification. The 5’ TTTGCAACTAGTATGGGGAGTAGCAAGAGCAAGC 3’ forward and 5’ AGAGAAGTTCGTGGCTCCGGATCCGGCCGTGCCGCTGGCCGAGTAGG 3’ reverse were used on the pCS2-ca Akt1 hum (a gift from Chia-Jung Shen) and the 5’ CCTACTCGGCCAGCGGCACGGCCGGATCCGGAGCCACGAACTTCTCT 3’ forward and 5’-CTATGACCATGATTACGCCAAG 3’ reverse primers onto the pCS2-mCherry-CAAX plasmid.

The caErk-P2A-mCherry cassette was generated by directed mutagenesis and PCR amplification. First, Erk cDNA was retrotranscribed using RNA extracted from 30 hpf embryos. RNA extraction was performed using RNeasy Mini kit (Cat. No.217004, Qiagen, France). Reverse Transcription (RT) was performed using High Capacity cDNA Reverse Transcription Kit (Part No 4368814, Applied Biosystems, UK), according to manufacturer’s instructions. Then, Erk cDNA was cloned into the pCS2 vector digested by EcoRI and XbaI enzymes. Three successive mutagenesis were performed using Q5 Polymerase (NEB) to create pCS2-ca-Erk (verified at all steps by Sanger sequencing). All primers used are listed below:

EcoRI-Erk2 fwd: 5’ CGATTCGAATTCAATGGCGACAGCTGCGGTTTCTGCCCC 3’.

XbaI-Erk2 rev: 5’CGAGACCTGAAGCCAGACAACCTGTTGCTCAACAC 3’.

ERK2/L84P fwd: CTGCGTGAGATTAAAATCCCGCTCCGCTTCAAGC.

ERK2/L84P rev: GCTTGAAGCGGAGCGGGATTTTAATCTCACGCAG.

ERK2/S162D fwd: CGAGACCTGAAGCCAGACAACCTGTTGCTCAACAC.

ERK2/S162D rev: GTGTTGAGCAACAGGTTGTCTGGCTTCAGGTCTCG.

ERK2/D330N fwd: CCTGGAGCAGTACTATGATCCTACAAATGAGCCTGTTGCTGAG.

ERK2/D330N rev: CTCAGCAACAGGCTCATTTGTAGGATCATAGTACTGCTCCAGG.

The 5’ TTTGCAACTAGTATGGCGACAGCTGCGGTTTC 3’ forward and 5’ AGAGAAGTTCGTGGCTCCGGATCCTGGTCTGTAGCCTGGCTGG 3’ reverse were used on the pCS2-ca Erk and the 5’ CCAGCCAGGCTACAGACCAGGATCCGGAGCCACGAACTTCTCT 3’ forward and 5’-CTATGACCATGATTACGCCAAG-3’ reverse primers onto the pCS2-mCherry-CAAX plasmid.

Similarly, the P2A-mcherry was amplified using 5’ ATGCCTGACTAGTGGATCCGGAGCCACGAACTTC 3’ and 5’-CTATGACCATGATTACGCCAAG 3’ reverse primers onto the pCS2-mCherry-CAAX plasmid. All the resulting PCR fragments were digested by SpeI and NotI before sub-cloning into the p5E-hb9 vector to generate all the phb9: constructs.

### Touch test assay

48 hpf embryos were pooled together and dechorionated at least 1 h before the assay. Fish were touched with the tip of forceps at the dorsal side of the hindbrain. A normal movement refers to an escape response in which the larvae moved to a distance at least 2 times its own body length, otherwise the escape response is considered reduced or abnormal (a circular movement was often observed in *MZrgs4*).

### Ly294002, U0126, Rapamycin and MHY1485 treatment

Embryos were incubated in either 10 μM of Ly294002 (Cell signaling, #9901), 20 μM of U0126 (Promega, #V1121) or a combination of both, 10 μM of Rapamycin (Sigma, #R0395) and 50 μM of MHY1485 (Sigma, #SML0810), all diluted in DMSO solution between 24 and 48 hpf. Controls were incubated in equivalent amount of DMSO solution (0.1%) during the same periods.

### Microinjections

Rgs4 and constitutive active forms of Akt or Erk mRNAs were synthesized using SP6 mMessage mMachine System after pCS2 vectors linearization with NotI and injected at 50 pg per embryo (for *rgs4* and *caErk*) and 10 pg (for *caAkt)*.

### Immunofluorescence

The following antibodies and dilutions were used: mouse anti-acetylated tubulin (Sigma; 1:500), mouse anti-HuC/D (Molecular probes; 1:500), rabbit anti-PH3 (Millipore; 1:500), mouse anti-znp1 (Developmental Studies Hybridoma Bank; 1:100). Primary antibodies were detected with appropriate secondary antibodies conjugated to either Alexa 488 or Alexa 568 (Molecular probes) at a 1:500 dilution. For immunostaining, embryos were fixed in 4% paraformaldehyde 1X PBS overnight at 4 °C, then washed with 1xPBS. Samples were then blocked with 0.5% triton in PBS and 10% sheep serum and then incubated with primary antibody overnight at 4 °C (diluted in PBS + 2% sheep serum). Larvae were then washed with PBS for a few hours and then incubated with secondary antibody in 0.5% triton in PBS and 2% sheep serum overnight at 4 °C. Embryos were incubated for 30 min in PBS triton containing 50 μg/ml of DAPI for nuclei counterstaining when needed. Stained larvae were then imaged with a Leica SP8 confocal microscope.

### Neural cell counting

Embryos were immunolabeled with HuC antibody (Molecular probes). Total neuronal (HuC+) cell numbers were counted from confocal z-series of the PLLg or spinal cord with an interval of 3 μm and was performed using Image J. Neurons were counted blindly by two independent researchers. The same applies to HuC−/DAPI and PH3+ cells.

### Motoneurons axonogenesis and branching analysis

Znp1 labeled embryos were imaged using a Sp8 Leica confocal at around 30 hpf. Axonal growth of the same four consecutive somites per embryo was assessed offline using manual tracing in image J. Axonal branching was assessed at 48 hpf using manual tracing in image J by analyzing znp1 images captured in z sections and projected in image J. Only fascicles ventral to the spinal cord of the same four consecutive somites per embryo were analyzed here.

### Head area and perimeter analysis

HuC/DAPI stained embryos were mounted dorsally and z stacks covering the telencephalon to hindbrain regions along the AP axis were captured. Z projections were generated (around 400 μm) and head and perimeter data were analyzed using image J (https://imagej.nih.gov/ij/).

### Western Blots

Proteins were extracted from pools of embryos with 10 μl lysis buffer (63 mM Tris HCl pH 6.8, glycerol 10% and SDS 3.5%, 10 mM Na_3_VO_4_ and cocktail of protease inhibitors) per embryo. Protein content was determined using the Pierce BCA protein assay. 30 μg proteins were loaded on gel. Western blots were performed according to standard methods using the following antibodies: mouse anti ß-actin (1/10,000; Sigma, A5441), anti-Erk and p-Erk (1/2500; Cell signaling, #4696 and #4370 respectively), rabbit anti-Akt and p-Akt (1/2500; Cell signaling, #9272 and #4060 respectively), anti-S6 and p-S6 (1/2000; Cell Signaling, #2317 and #2215 respectively) , rabbit anti ß-tubulin (1/5000; Abcam, ab18251), rabbit anti-GAPDH (1/500; GeneTex, GTX124503) and HRP conjugated secondary antibodies (1/10,000; Sigma, A2304 and A5420). Densitometry quantification (N = 4 samples) was done using ImageJ software (NIH).

### Neural cells culture

48 hpf WT and *MZrgs4* embryos were bleached for 2 min with 0.005% hypochlorite, washed three times with water and anesthetized with 0.03% tricaine before dissection as described in^38^. 35,000 cells per well (24 well plates) were incubated overnight at 29° in zebrafish complete media composed of L-15 (Gibco), supplemented with 2.5 mM glutamax (Fisher Scientific), 15 ng/mL EGF (Fisher Scientific), 10% fetal bovine serum and 5% zebrafish extract (prepared as detailed in^61^). Cells were lysed the next day with 30µL of RIPA buffer + 10 mM Na3VO4 and cocktail of protease inhibitors to be used for WB.

### Quantitative RT-qPCR

For qPCR analysis, RNA extraction was performed using RNeasy Mini kit (Cat. No.217004, Qiagen, France) according to manufactures' instructions from total zebrafish embryos. Reverse Transcription (RT) was performed using High Capacity cDNA Reverse Transcription Kit (Part No 4368814, Applied Biosystems, UK), according to manufacturer’s instructions.

Primers used were:

Rgs4 fwd 5′-GTGTAAAGGGCTTGCTGCTCTT-3′.

Rgs4 rev 5′-GTTTCGGCAGGAGTGATTCTGT-3′.

Elfa fwd 5’-CTTCTCAGGCTGACTGTGC-3’.

Elfa rev 5’-CCGCTAGCATTACCCTCC-3’.

Neurod1 fwd 5’-AAGCTTTCAACACACCCTAGAGTTC-3′.

Neurod1 rev 5’-ATCGTCGTCTTCCATATCGTTGA-3′.

Neurog1 fwd 5’-CGCACACGGATGATGAAGACT-3′.

Neurog1 rev 5’-CGGTTCTTCTTCACGACGTG-3′.

### Acridine orange staining

Embryos were anesthetised with 0.03% tricaine and incubated in 5 μM acridine orange for 20 min in the dark. After wash, they were embedded in 1.5% low melting point agarose, and imaged with a Leica SP8 confocal microscope.

### Statistical analysis

Means and standard deviations were calculated with Graph Pad Prism 7. All data was first tested for normal distribution using the ShapiroWilk normality test combined with D'Agostino & Pearson normality test. All experiments with only two groups and one dependent variable were compared using an unpaired *t*-test with Welch’s correction if they passed normality test; if not, groups were compared with the nonparametric Mann–Whitney test. Statistically significant differences were determined using one-way ANOVA for all experiments with more than two groups but only one dependent variable. Error bars depict standard errors of the mean (SEM). ns, p > 0.05; *, p ≤ 0.05; **, p ≤ 0.01; ***, p ≤ 0.001; ****, p ≤ 0.0001. n represents the number of embryos.

## Supplementary Information

Supplementary Information 1.

Supplementary Information 2.

Supplementary Information 3.

Supplementary Information 4.

Supplementary Information 5.

Supplementary Information 6.

Supplementary Information 7.

Supplementary Information 8.

## References

[1] Bohm A, Gaudet R, Sigler PB (1997). Structural aspects of heterotrimeric G-protein signaling. Curr. Opin. Biotechnol..

[2] Brandt DR, Ross EM (1985). GTPase activity of the stimulatory GTP-binding regulatory protein of adenylate cyclase, Gs. Accumulation and turnover of enzyme-nucleotide intermediates. J. Biol. Chem..

[3] Higashijima T, Ferguson KM, Sternweis PC, Smigel MD, Gilman AG (1987). Effects of Mg2+ and the beta gamma-subunit complex on the interactions of guanine nucleotides with G proteins. J. Biol. Chem..

[4] Wall MA, Posner BA, Sprang SR (1998). Structural basis of activity and subunit recognition in G protein heterotrimers. Structure.

[5] Chan HCS, Li Y, Dahoun T, Vogel H, Yuan S (2019). New binding sites, new opportunities for GPCR drug discovery. Trends Biochem. Sci..

[6] Weis WI, Kobilka BK (2018). The molecular basis of G protein-coupled receptor activation. Annu. Rev. Biochem..

[7] Evanko DS, Thiyagarajan MM, Siderovski DP, Wedegaertner PB (2001). Gbeta gamma isoforms selectively rescue plasma membrane localization and palmitoylation of mutant Galphas and Galphaq. J. Biol. Chem..

[8] Robillard L, Ethier N, Lachance M, Hebert TE (2000). Gbetagamma subunit combinations differentially modulate receptor and effector coupling in vivo. Cell Signal.

[9] Blumer KJ (2004). Vision: The need for speed. Nature.

[10] Doupnik CA, Davidson N, Lester HA, Kofuji P (1997). RGS proteins reconstitute the rapid gating kinetics of gbetagamma-activated inwardly rectifying K+ channels. Proc. Natl. Acad. Sci. U.S.A..

[11] He W, Cowan CW, Wensel TG (1998). RGS9, a GTPase accelerator for phototransduction. Neuron.

[12] Bansal G, Druey KM, Xie Z (2007). R4 RGS proteins: regulation of G-protein signaling and beyond. Pharmacol. Ther..

[13] Hurst JH, Hooks SB (2009). Regulator of G-protein signaling (RGS) proteins in cancer biology. Biochem. Pharmacol..

[14] Kimple AJ, Bosch DE, Giguere PM, Siderovski DP (2011). Regulators of G-protein signaling and their Galpha substrates: Promises and challenges in their use as drug discovery targets. Pharmacol. Rev..

[15] Lee JK, Bou Dagher J (2016). Regulator of G-protein signaling (RGS)1 and RGS10 proteins as potential drug targets for neuroinflammatory and neurodegenerative diseases. AAPS J..

[16] Neubig RR, Siderovski DP (2002). Regulators of G-protein signalling as new central nervous system drug targets. Nat. Rev. Drug Discov..

[17] O'Brien JB, Wilkinson JC, Roman DL (2019). Regulator of G-protein signaling (RGS) proteins as drug targets: Progress and future potentials. J. Biol. Chem..

[18] Willars GB (2006). Mammalian RGS proteins: Multifunctional regulators of cellular signalling. Semin Cell Dev Biol.

[19] Erdely HA (2004). Regional expression of RGS4 mRNA in human brain. Eur. J. Neurosci..

[20] Grillet N, Dubreuil V, Dufour HD, Brunet JF (2003). Dynamic expression of RGS4 in the developing nervous system and regulation by the neural type-specific transcription factor Phox2b. J. Neurosci..

[21] Zhang S (1998). RGS3 and RGS4 are GTPase activating proteins in the heart. J. Mol. Cell Cardiol..

[22] Berman DM, Wilkie TM, Gilman AG (1996). GAIP and RGS4 are GTPase-activating proteins for the Gi subfamily of G protein alpha subunits. Cell.

[23] Gold SJ (2003). Regulation of RGS proteins by chronic morphine in rat locus coeruleus. Eur. J. Neurosci..

[24] Grillet N (2005). Generation and characterization of Rgs4 mutant mice. Mol. Cell. Biol..

[25] Bakker SC (2007). The PIP5K2A and RGS4 genes are differentially associated with deficit and non-deficit schizophrenia. Genes Brain Behav..

[26] Bowden NA, Scott RJ, Tooney PA (2007). Altered expression of regulator of G-protein signalling 4 (RGS4) mRNA in the superior temporal gyrus in schizophrenia. Schizophr Res..

[27] Chowdari KV (2008). Linkage disequilibrium patterns and functional analysis of RGS4 polymorphisms in relation to schizophrenia. Schizophr Bull..

[28] Cheng YC (2013). Zebrafish rgs4 is essential for motility and axonogenesis mediated by Akt signaling. Cell Mol Life Sci..

[29] Wu C, Zeng Q, Blumer KJ, Muslin AJ (2000). RGS proteins inhibit Xwnt-8 signaling in Xenopus embryonic development. Development.

[30] Leone AM, Errico M, Lin SL, Cowen DS (2000). Activation of extracellular signal-regulated kinase (ERK) and Akt by human serotonin 5-HT(1B) receptors in transfected BE(2)-C neuroblastoma cells is inhibited by RGS4. J. Neurochem..

[31] Weiler M (2013). Suppression of proinvasive RGS4 by mTOR inhibition optimizes glioma treatment. Oncogene.

[32] Xue X, Wang L, Meng X, Jiao J, Dang N (2017). Regulator of G protein signaling 4 inhibits human melanoma cells proliferation and invasion through the PI3K/AKT signaling pathway. Oncotarget.

[33] Fontenas L (2016). Neuronal Ndrg4 is essential for nodes of Ranvier organization in Zebrafish. PLoS Genet..

[34] Mikdache A (2019). Elmo1 function, linked to Rac1 activity, regulates peripheral neuronal numbers and myelination in zebrafish. Cell Mol. Life Sci..

[35] Giustiniani J (2014). Immunophilin FKBP52 induces Tau-P301L filamentous assembly in vitro and modulates its activity in a model of tauopathy. Proc. Natl. Acad. Sci. U.S.A..

[36] Chitnis AB, Nogare DD, Matsuda M (2012). Building the posterior lateral line system in zebrafish. Dev. Neurobiol..

[37] Montgomery J, Carton G, Voigt R, Baker C, Diebel C (2000). Sensory processing of water currents by fishes. Philos. Trans. R. Soc. Lond. B Biol. Sci..

[38] Patel BB (2019). Isolation and culture of primary embryonic zebrafish neural tissue. J. Neurosci. Methods.

[39] Finkielsztein A, Kelly GM (2009). Altering PI3K-Akt signalling in zebrafish embryos affects PTEN phosphorylation and gastrulation. Biol. Cell.

[40] Hawkins TA, Cavodeassi F, Erdelyi F, Szabo G, Lele Z (2008). The small molecule Mek1/2 inhibitor U0126 disrupts the chordamesoderm to notochord transition in zebrafish. BMC Dev. Biol..

[41] Hong CC, Peterson QP, Hong JY, Peterson RT (2006). Artery/vein specification is governed by opposing phosphatidylinositol-3 kinase and MAP kinase/ERK signaling. Curr. Biol..

[42] Vlahos CJ, Matter WF, Hui KY, Brown RF (1994). A specific inhibitor of phosphatidylinositol 3-kinase, 2-(4-morpholinyl)-8-phenyl-4H-1-benzopyran-4-one (LY294002). J. Biol. Chem..

[43] Choi YJ (2012). Inhibitory effect of mTOR activator MHY1485 on autophagy: suppression of lysosomal fusion. PLoS ONE.

[44] Wang X (2019). Tanshinone IIA restores dynamic balance of autophagosome/autolysosome in doxorubicin-induced cardiotoxicity via targeting Beclin1/LAMP1. Cancers (Basel)..

[45] Yu JS, Cui W (2016). Proliferation, survival and metabolism: the role of PI3K/AKT/mTOR signalling in pluripotency and cell fate determination. Development.

[46] Chadwick ML (2018). Combined mTOR and MEK inhibition is an effective therapy in a novel mouse model for angiosarcoma. Oncotarget.

[47] Sunayama J (2010). Crosstalk between the PI3K/mTOR and MEK/ERK pathways involved in the maintenance of self-renewal and tumorigenicity of glioblastoma stem-like cells. Stem Cells.

[48] Fassier C (2018). Motor axon navigation relies on Fidgetin-like 1-driven microtubule plus end dynamics. J. Cell Biol..

[49] Fassier C (2010). Zebrafish atlastin controls motility and spinal motor axon architecture via inhibition of the BMP pathway. Nat. Neurosci..

[50] Guillon E, Bretaud S, Ruggiero F (2016). Slow muscle precursors lay down a collagen XV matrix fingerprint to guide motor axon navigation. J. Neurosci..

[51] Gwee SSL (2018). Aurora kinase B regulates axonal outgrowth and regeneration in the spinal motor neurons of developing zebrafish. Cell Mol. Life Sci..

[52] le Hao T (2017). HuD and the survival motor neuron protein interact in motoneurons and are essential for motoneuron development, function, and mRNA regulation. J. Neurosci..

[53] Oprisoreanu, A. M. *et al.* Interaction of axonal chondrolectin with collagen XIXa1 is necessary for precise neuromuscular junction formation. *Cell Rep.* **29**, 1082–1098 e1010. 10.1016/j.celrep.2019.09.033 (2019). 10.1016/j.celrep.2019.09.033 31665626

[54] Kurabayashi N, Nguyen MD, Sanada K (2013). The G protein-coupled receptor GPRC5B contributes to neurogenesis in the developing mouse neocortex. Development.

[55] Qiu R, Wang J, Tsark W, Lu Q (2010). Essential role of PDZ-RGS3 in the maintenance of neural progenitor cells. Stem Cells.

[56] Novorol C (2013). Microcephaly models in the developing zebrafish retinal neuroepithelium point to an underlying defect in metaphase progression. Open Biol..

[57] Reilly ML (2019). Loss-of-function mutations in KIF14 cause severe microcephaly and kidney development defects in humans and zebrafish. Hum. Mol. Genet..

[58] Ahlers-Dannen KE, Spicer MM, Fisher RA (2020). RGS proteins as critical regulators of motor function and their implications in Parkinson's disease. Mol. Pharmacol..

[59] Park HC (2000). Analysis of upstream elements in the HuC promoter leads to the establishment of transgenic zebrafish with fluorescent neurons. Dev. Biol..

[60] Varshney GK (2015). High-throughput gene targeting and phenotyping in zebrafish using CRISPR/Cas9. Genome Res..

[61] Kinikoglu B, Kong Y, Liao EC (2014). Characterization of cultured multipotent zebrafish neural crest cells. Exp. Biol. Med. (Maywood).

