# Supplementary material for: Rgs4 is a regulator of mTOR activity required for motoneuron axon outgrowth and neuronal development in zebrafish
Source: Sci Rep. 2021 Jun 25;11:13338. doi: 10.1038/s41598-021-92758-z (PMC8233358; doi:10.1038/s41598-021-92758-z)
Supplement: Supplementary file 1 — Supplementary Information 1. [file 41598_2021_92758_MOESM1_ESM.docx]

**Supplementary Figure 1.** RT-qPCR analysis of *MZrgs4* embryos shows a significant decrease in the expression of *rgs4* mRNA in comparison to WT embryos at 48 hpf. Expression was normalized to *elfa* expression.

**Supplementary Figure 2. *MZrgs4* embryos show a decrease in *neurod1* mRNA expression and normal neural cell death.** (A) RT-qPCR showing a significant decrease in the expression of *neurod1* at 48 hpf in *MZrgs4* mutants. Expression was normalized to *elfa* expression. (B,C) AO staining showing apoptotic cells in the PLLg in WT (B) and *MZrgs4* (C) at 48 hpf. Dashed lines represent the PLLg. Scale bar = 20μm. (D) Quantification of the number of AO positive cells in the PLLg in WT (average of 0.56±0.18, n=16) and *MZrgs4* embryos (average of 0.66±0.18, n=18) at 48 hpf. (E,F) AO staining showing apoptotic cells in the spinal cord in WT (E) and *MZrgs4* (F) at 48 hpf. Dashed lines represent the spinal cord. Scale bar = 25 μm. (G) Quantification of the number of AO positive cells in the spinal cord in WT (average of 1±0.26, n=11) and *MZrgs4* embryos (average of 1.16±0.27, n=12). ns, non significant. AO, Acridine Orange.

**Supplementary Figure 3.** Original Immunoblotting of lysates from zebrafish embryos at 20 hpf related to Figures 5 and 7.

**Supplementary Figure 4.** Original Immunoblotting of lysates from zebrafish embryos at 48 hpf related to Figures 5 and 7.

**Supplementary Figure 5.** Original Immunoblotting of neural lysates from zebrafish embryos at 48 hpf related to Figures 5 and 7.

**Supplementary Figure 6. Akt and ERK signaling pathways contribute to neuronal development** (A) Quantification of the number of neurons in the PLLg in WT (average of 55.81±1.06, n=16), Ly294002 (average of 35.42±1.24, n=12), U0126 (average of 35.50±1.40, n=12) and Ly294002+U0126 (average of 39.90±0.70, n=10) treated embryos at 48 hpf. (B-D) Z projections of whole-mount immunostaining for Znp1 labeling the ramifications of motoneurons in drug treated embryos Ly294002 (B), U0126 (C) and Ly294002+U0126 (D) at 48 hpf. (E) Quantification of the number of ramifications per motoneuron in WT (average of 21.05±1.10, 40 motoneurons, n=12), Ly294002 (average of 11.79±0.49, 47 motoneurons, n=14), U0126 (average of 12.50±0.80, 38 motoneurons, n=12), Ly294002+U0126 (average of 7.16±0.68, 40 motoneurons, n=12) at 48 hpf.

**Supplementary Figure 7. mTOR activity is required for neuronal development and PI3K/Akt and MEK/Erk activities are linked to mTOR signaling in *MZrgs4* embryos**

(A) Quantification of the number of neurons in the PLLg in WT (average of 56.23±1.17, n=13), WT+Rapamycin (average of 35.58±0.98, n=12), *MZrgs4* (average of 28.10±1.01, n=20) and *MZrgs4*+MHY (average of 36±0.87, n=17) at 48 hpf. (B-E) Z projections of whole-mount immunostaining for Znp1 labeling the ramifications of motoneurons in WT (B), Rapamycin (C), *MZrgs4* (D) and *MZrgs4*+MHY (E) at 48 hpf. Scale bar = 20μm. (F) Quantification of the number of ramifications per motoneuron in WT (average of 20.64±1.10, 32 motoneurons, n=11), WT+Rapamycin (average of 11.83±0.67, 36 motoneurons, n=12), *MZrgs4* (average of 9.40±0.90, 50 motoneurons, n=16) and *MZrgs4*+MHY (average of 15.33±2.58, 46 motoneurons, n=15) at 48 hpf. (G) Quantification of the number of neurons in the PLLg in *MZrgs4* (average of 28.30±1.26, n=20), *MZrgs4*+caAkt (average of 55±1.36, n=22) and *MZrgs4*+caAkt+Rapamycin (average of 43.25±0.95, n=12) at 48 hpf. (H) Quantification of the number of neurons in the PLLg in *MZrgs4* (average of 28.35±1.25, n=20), *MZrgs4*+caErk (average of 48.32±0.83, n=25) and *MZrgs4*+caErk+Rapamycin (average of 38.43±1.18, n=14) at 48 hpf. (I-L) Z projections of whole-mount immunostaining for Znp1 labeling the ramifications of motoneurons in WT (I), *MZrgs4* (J), *MZrgs4*+caAkt+Rapamycin (K) and *MZrgs4*+caErk+Rapamycin (L) at 48 hpf. (M) Quantification of the number of ramifications per motoneuron in *MZrgs4* (average of 9.11±0.8, 44 motoneurons, n=18), *MZrgs4*+caAkt (average of 19.83±1.11, 36 motoneurons, n=12) and *MZrgs4*+caAkt+Rapamycin (average of 7.84±0.55, 34 motoneurons, n=13) at 48 hpf. (N) Quantification of the number of ramifications per motoneuron in *MZrgs4* (average of 9.05±0.76, 40 motoneurons, n=19), *MZrgs4*+caErk (average of 19.79±0.86, 38 motoneurons, n=14) and *MZrgs4*+caErk+Rapamycin (average of 7.35±0.75, 36 motoneurons, n=14) at 48 hpf.
