# Supplementary figures and images for: Rgs4 is a regulator of mTOR activity required for motoneuron axon outgrowth and neuronal development in zebrafish
Source: Sci Rep. 2021 Jun 25;11:13338. doi: 10.1038/s41598-021-92758-z (PMC8233358; doi:10.1038/s41598-021-92758-z)

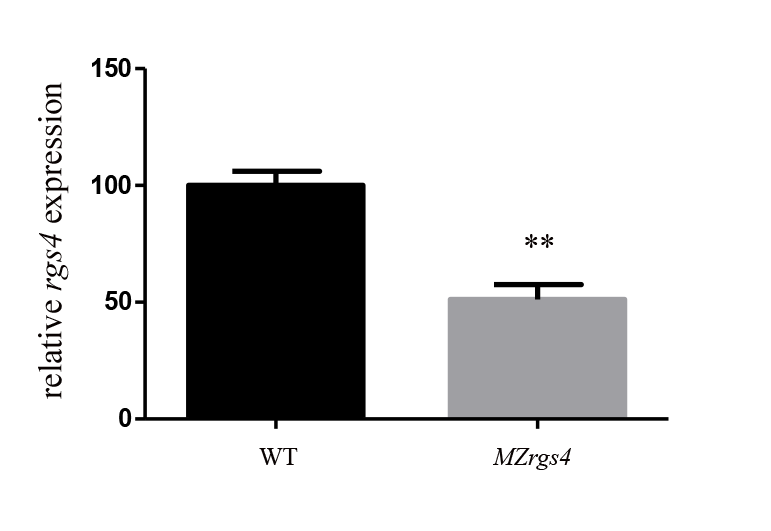

Supplement: Supplementary file 2 — Supplementary Information 2. [file 41598_2021_92758_MOESM2_ESM.tif]

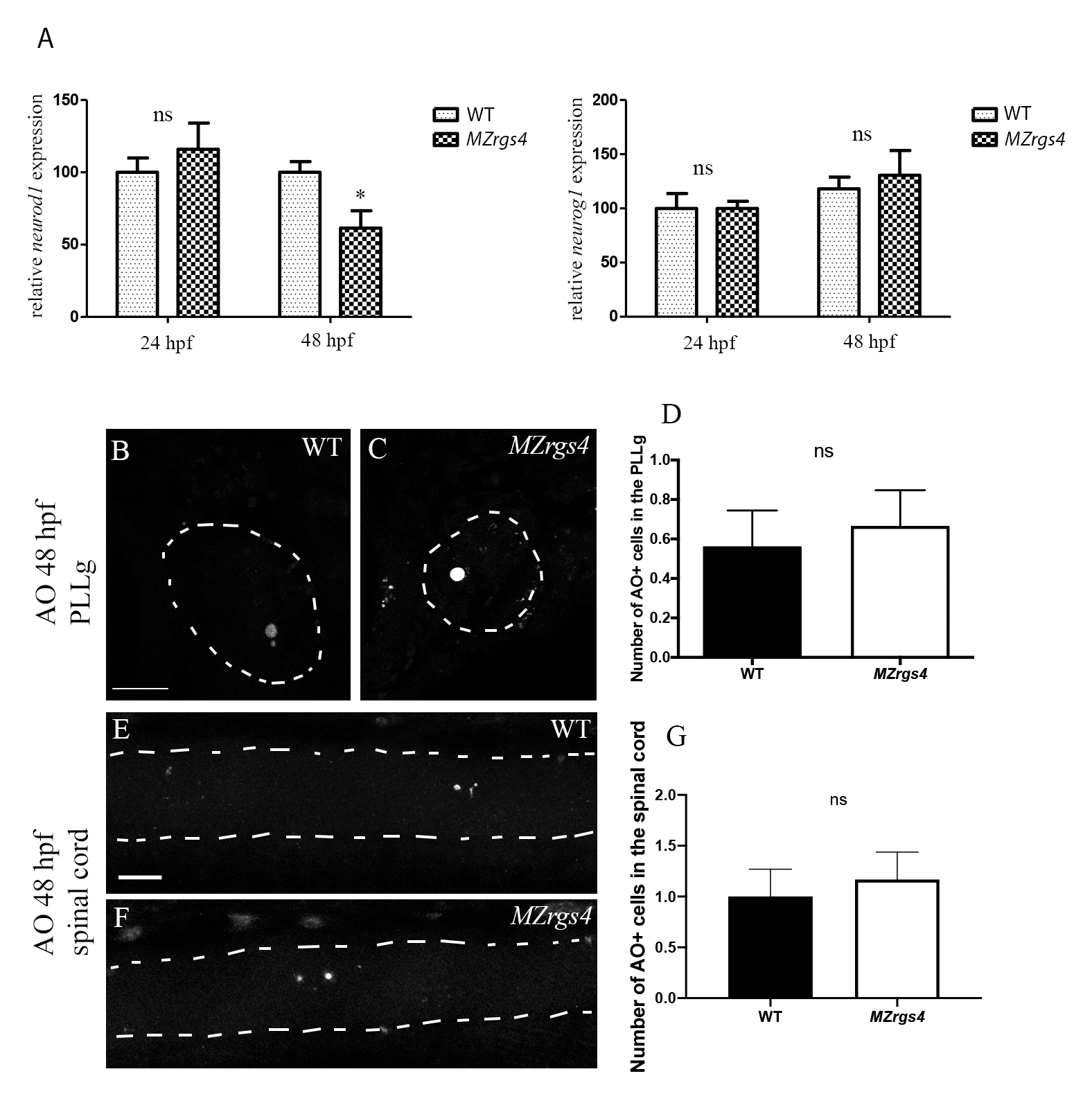

Supplement: Supplementary file 3 — Supplementary Information 3. [file 41598_2021_92758_MOESM3_ESM.tif]

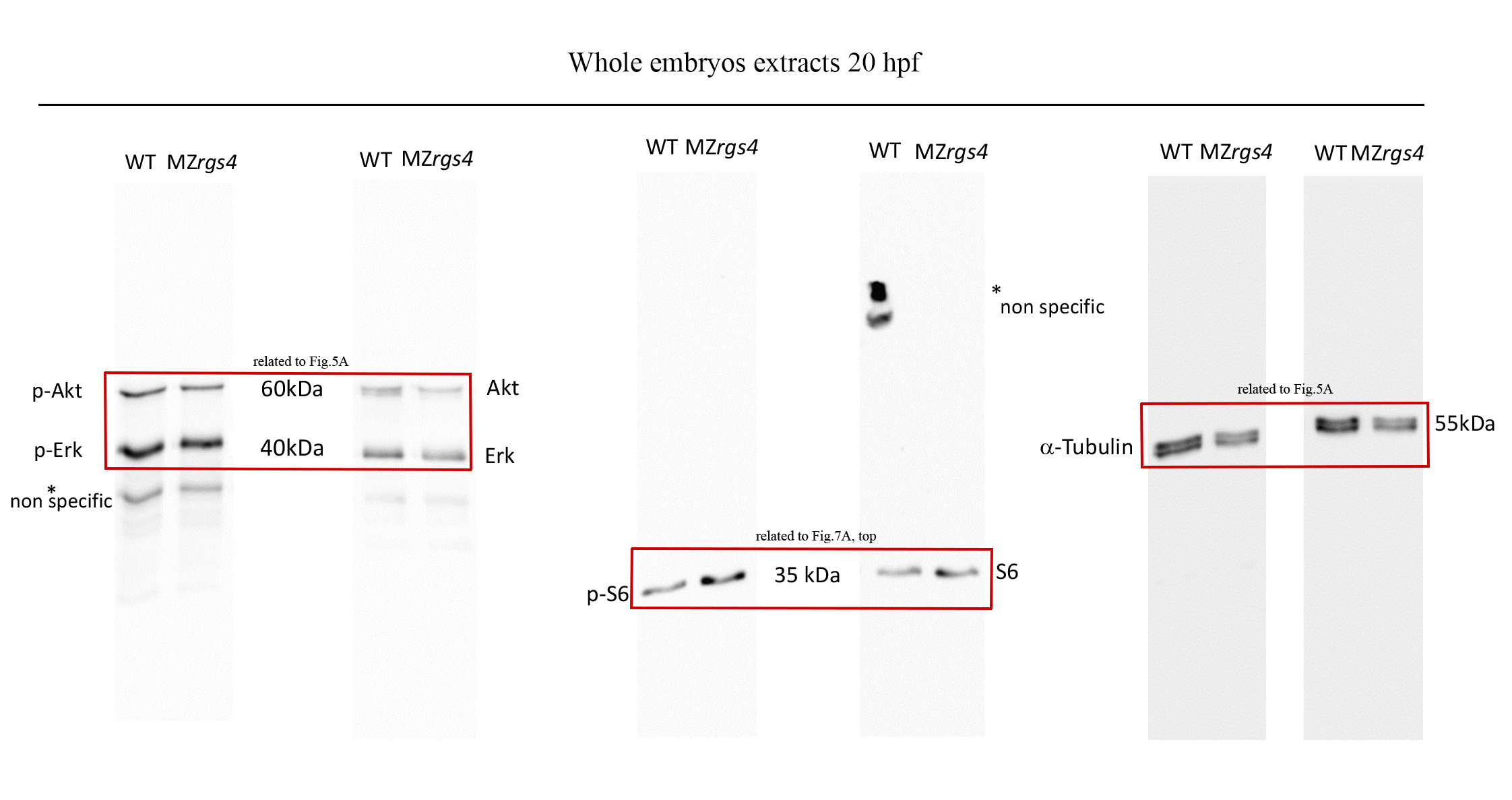

Supplement: Supplementary file 4 — Supplementary Information 4. [file 41598_2021_92758_MOESM4_ESM.tif]

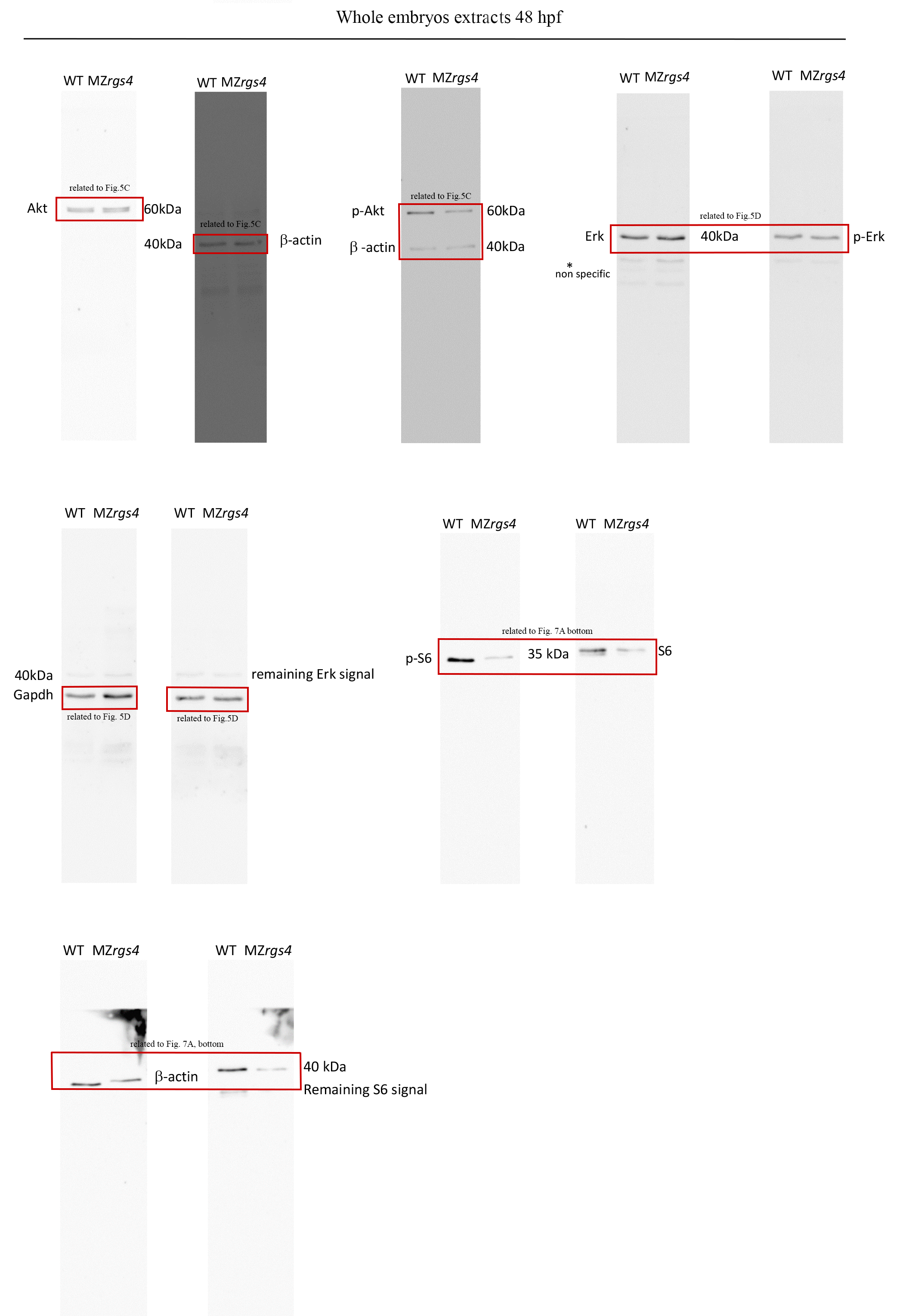

Supplement: Supplementary file 5 — Supplementary Information 5. [file 41598_2021_92758_MOESM5_ESM.tif]

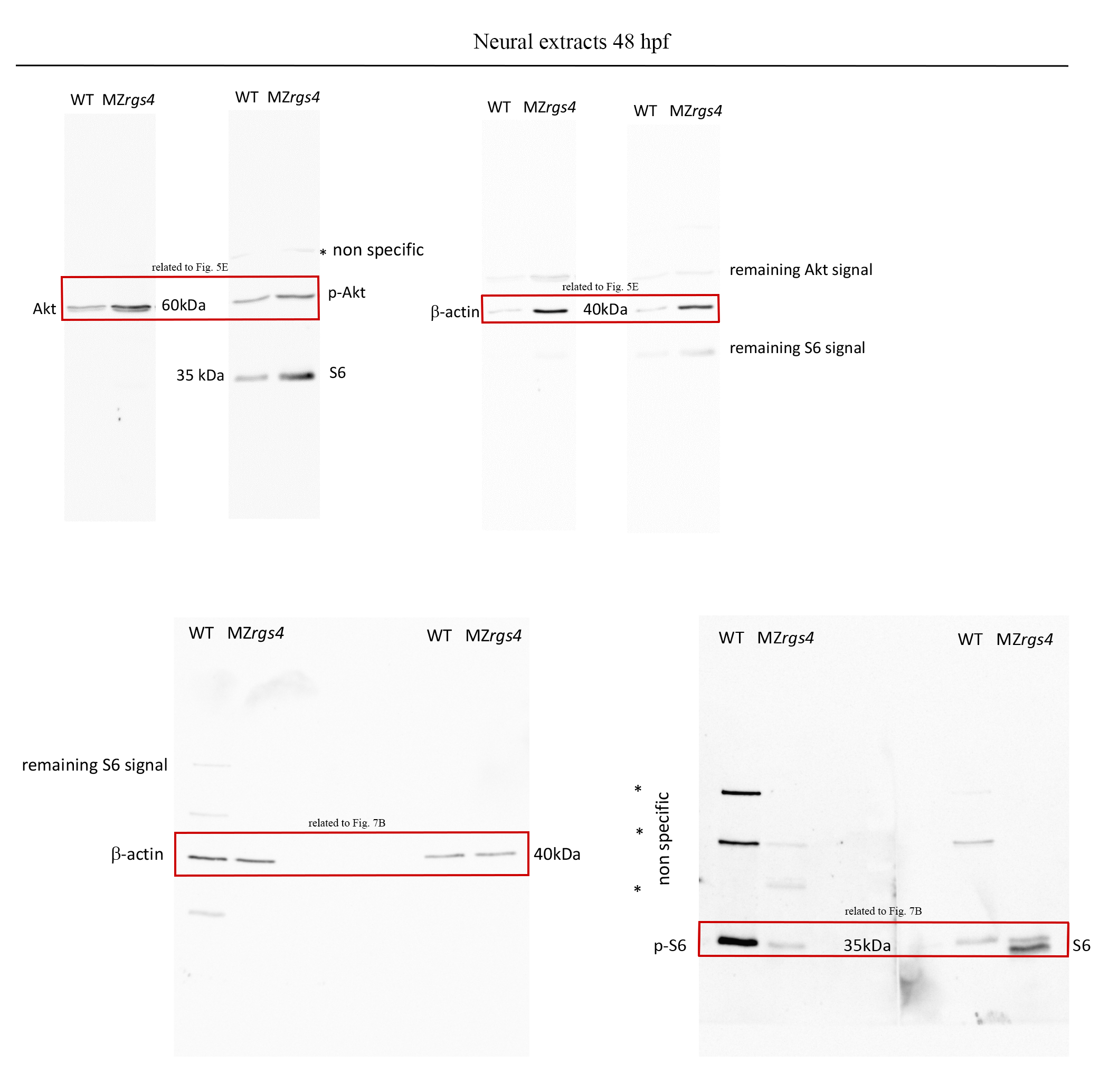

Supplement: Supplementary file 6 — Supplementary Information 6. [file 41598_2021_92758_MOESM6_ESM.tif]

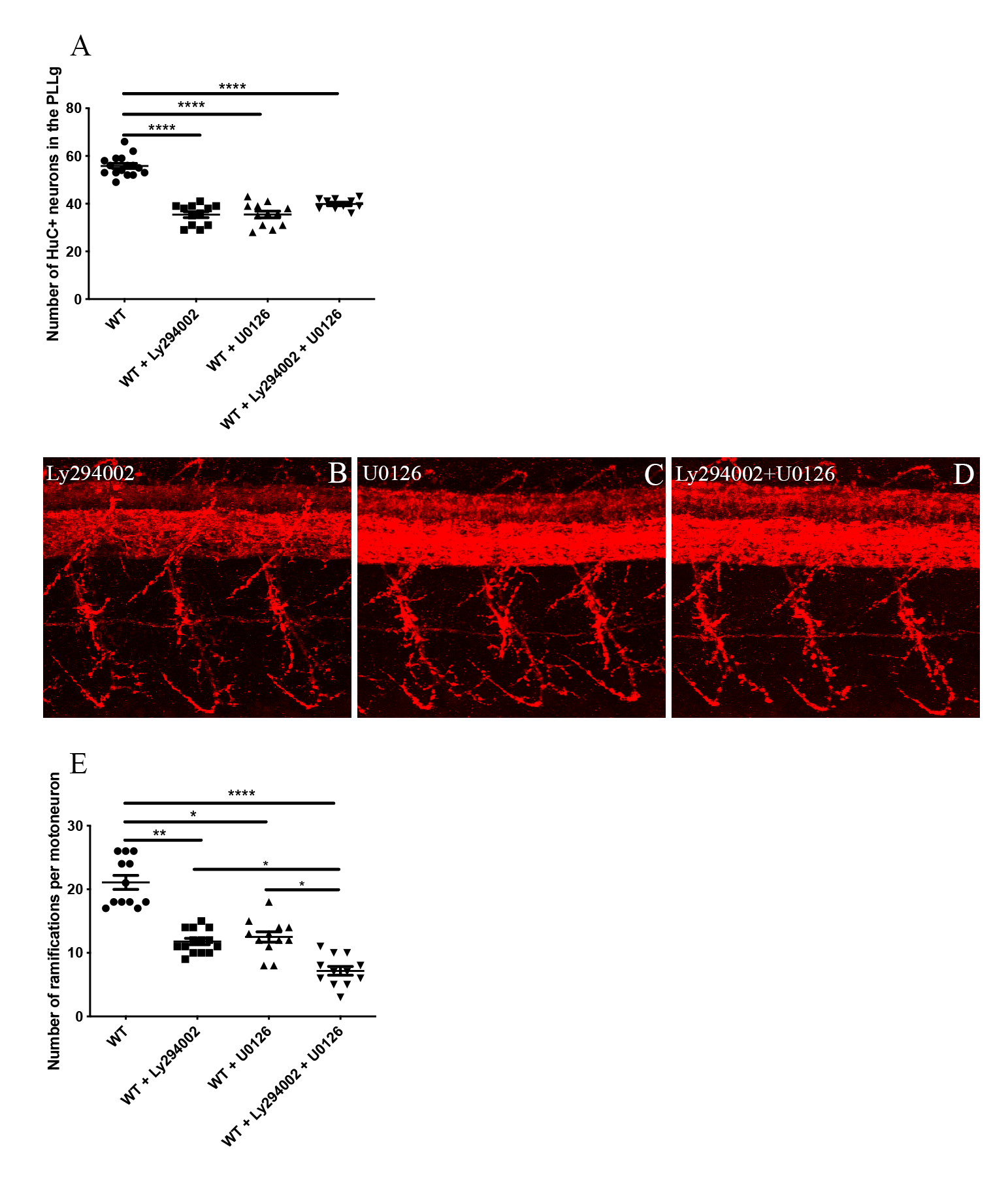

Supplement: Supplementary file 7 — Supplementary Information 7. [file 41598_2021_92758_MOESM7_ESM.tif]

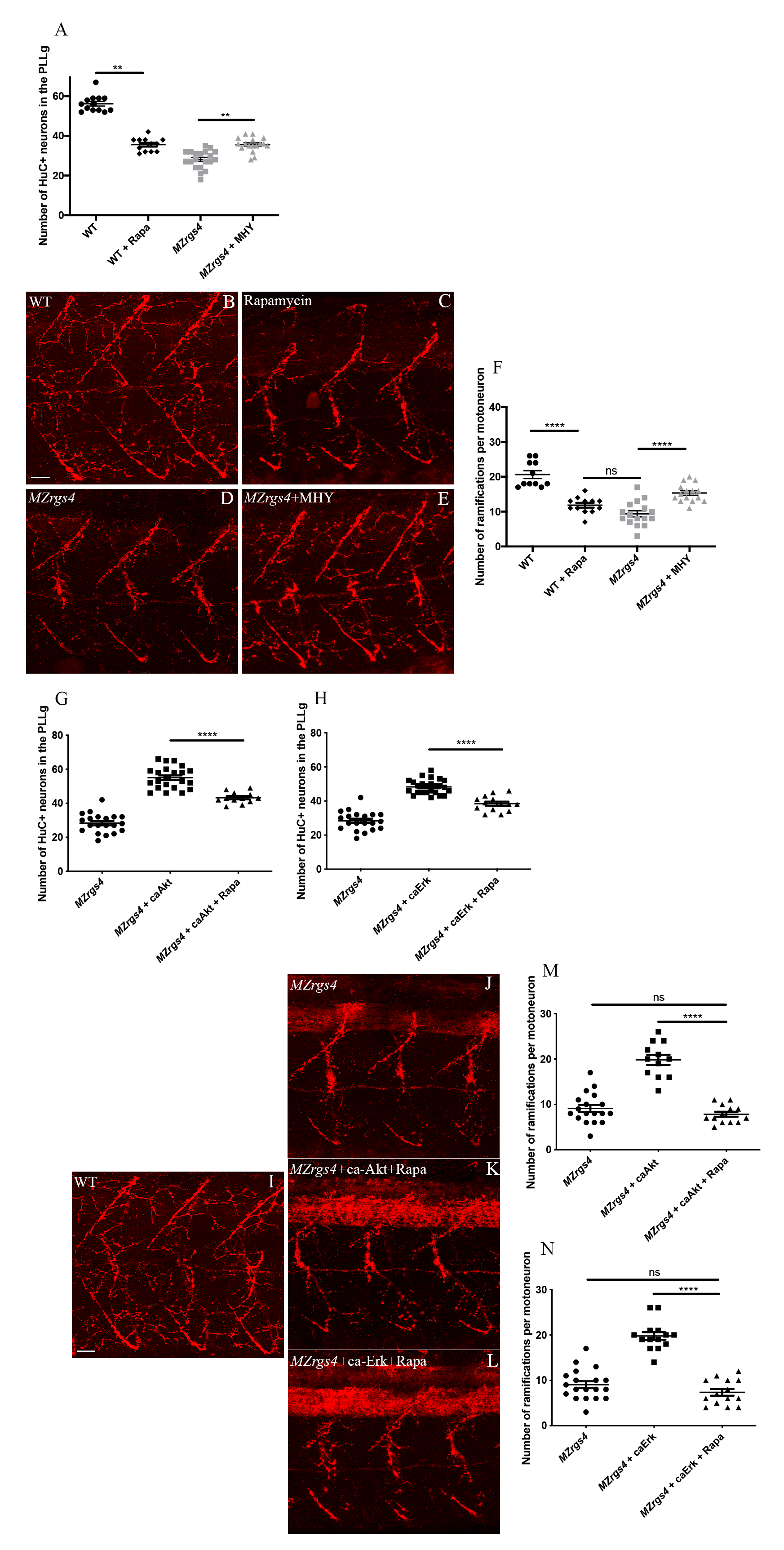

Supplement: Supplementary file 8 — Supplementary Information 8. [file 41598_2021_92758_MOESM8_ESM.tif]
